# Hepatic steatosis risk is partly driven by increased de novo lipogenesis following carbohydrate consumption

**DOI:** 10.1186/s13059-018-1439-8

**Published:** 2018-06-20

**Authors:** Francis W. B. Sanders, Animesh Acharjee, Celia Walker, Luke Marney, Lee D. Roberts, Fumiaki Imamura, Benjamin Jenkins, Jack Case, Sumantra Ray, Samuel Virtue, Antonio Vidal-Puig, Diana Kuh, Rebecca Hardy, Michael Allison, Nita Forouhi, Andrew J. Murray, Nick Wareham, Michele Vacca, Albert Koulman, Julian L. Griffin

**Affiliations:** 1 MRC Human Nutrition Research, Fulbourn Road, Cambridge, UK; 2 Department of Biochemistry and the Cambridge Systems Biology Centre, University of Cambridge, The Sanger Building, 80 Tennis Court Road, Cambridge, CB2 1GA UK; 3 Current address: Leeds Institute of Cardiovascular and Metabolic Medicine, University of Leeds, Leeds, UK; 4 MRC Epidemiology, University of Cambridge, Addenbrooke’s Hospital, Cambridge, UK; 5 NNEdPro Global Centre for Nutrition and Health, Cambridge, UK; 6 Institute of Metabolic Science, University of Cambridge, Cambridge, UK; 7 MRC Unit for Lifelong Health and Ageing at UCL, London, UK; 8 Liver Unit, Department of Medicine, Cambridge Biomedical Research Centre, Cambridge University Hospitals NHS Foundation Trust, Cambridge, UK; 9 Department of Physiology, Development and Neuroscience, University of Cambridge, Cambridge, UK

**Keywords:** Non-alcoholic fatty liver disease, Direct infusion mass spectrometry, De novo lipogenesis, Triacylglycerols, Triglycerides

## Abstract

**Background:**

Diet is a major contributor to metabolic disease risk, but there is controversy as to whether increased incidences of diseases such as non-alcoholic fatty liver disease arise from consumption of saturated fats or free sugars. Here, we investigate whether a sub-set of triacylglycerols (TAGs) were associated with hepatic steatosis and whether they arise from de novo lipogenesis (DNL) from the consumption of carbohydrates.

**Results:**

We conduct direct infusion mass spectrometry of lipids in plasma to study the association between specific TAGs and hepatic steatosis assessed by ultrasound and fatty liver index in volunteers from the UK-based Fenland Study and evaluate clustering of TAGs in the National Survey of Health and Development UK cohort. We find that TAGs containing saturated and monounsaturated fatty acids with 16–18 carbons are specifically associated with hepatic steatosis. These TAGs are additionally associated with higher consumption of carbohydrate and saturated fat, hepatic steatosis, and variations in the gene for protein phosphatase 1, regulatory subunit 3b (PPP1R3B), which in part regulates glycogen synthesis. DNL is measured in hyperphagic *ob/ob* mice, mice on a western diet (high in fat and free sugar) and in healthy humans using stable isotope techniques following high carbohydrate meals, demonstrating the rate of DNL correlates with increased synthesis of this cluster of TAGs. Furthermore, these TAGs are increased in plasma from patients with biopsy-confirmed steatosis.

**Conclusion:**

A subset of TAGs is associated with hepatic steatosis, even when correcting for common confounding factors. We suggest that hepatic steatosis risk in western populations is in part driven by increased DNL following carbohydrate rich meals in addition to the consumption of saturated fat.

**Electronic supplementary material:**

The online version of this article (10.1186/s13059-018-1439-8) contains supplementary material, which is available to authorized users.

## Background

Hypertriglyceridemia is a substantial co-morbidity associated with non-alcoholic fatty liver disease (NAFLD), cardiovascular disease (CVD), type 2 diabetes mellitus (T2DM), hypertension and cancer [1–4]. Triacylglycerols (also called triglycerides [TAGs]) are the largest energy store in the body and are transported from their major stores in adipose tissue to sites with a high metabolic demand, in particular slow-twitch skeletal muscle, heart and liver [5]. Diet has a major impact on TAG composition, total fatty acid intake [6, 7] and the synthesis of new fatty acids (FAs) in the liver by de novo lipogenesis (DNL) [8]. In the clinic, blood plasma TAG concentrations are measured by biochemical assay, but such assays do not discriminate between different TAG species that contribute to the total, limiting diagnostic and mechanistic interpretation.

Liquid chromatography mass spectrometry (LC-MS) separates TAG species by hydrophobicity and molecular mass [9]. Using such an approach, Rhee et al. reported positive associations between plasma TAG(44:1), TAG(46:1), TAG(48:1) and TAG(48:0) and the relative risk of developing T2DM in the Framingham Heart Study independent of age and sex [10]. Similarly, in the Bruneck cohort (Austria), TAG(54:2) was associated with increased risk of CVD [11]. Specific TAGs have also been associated with both pre-diabetes and T2DM, and have been shown to improve predictions of T2DM when used alongside classic risk factors such as HbA1c and total TAGs [12, 13]. Kotronen et al. examining TAG species in individuals across a broad ranges of insulin sensitivity hypothesised that circulating concentrations of specific TAGs (TAG(16:0/16:0/18:1) and TAG(16:0/18:1/18:0)) may be better predictors of the homeostatic model assessment of insulin resistance (HOMA-IR) than total TAG blood content [14], while TAGs containing unsaturated essential FAs were negatively associated with IR. Across these studies, the TAG species have in common relative enrichments for palmitate, stearate and oleate fatty acids, and studies using gas chromatography (GC)-MS have also reported associations between these FAs from total lipid extracts and increased risk of developing T2DM [15].

Although hepatic DNL is traditionally believed to contribute minimally to the total lipid pool in humans, increased rates of DNL have been described in both NAFLD and rare forms of IR and metabolic disease [16]. Semple et al. used deuterium labelled water to directly investigate DNL in those with either insulin signalling defects or lipodystrophy, describing increased rates of DNL with IR [17]. In addition, the plasma content of palmitoleic acid (C16:1 n-7) has been shown to be a sensitive marker for NAFLD [18]. Using direct infusion (DI)-MS, we demonstrated that individuals with lipodystrophy have increased TAGs selectively enriched for palmitate, stearate and oleate and these TAGs are predominantly found in VLDL [19]. In lipodystrophy, where adipose tissue depots are either totally or partially lacking, post-prandial glucose is metabolised in the liver, increasing hepatic DNL when glycogen reserves are replete. Furthermore, these patients have severe, peripheral IR and hyperinsulinemia, further increasing hepatic DNL when glucose is in excess. Here, we have conducted an observational epidemiological analysis, two murine studies, and a human feeding experiment to characterise the determinants of TAG species, and in particular how these TAGs are related to DNL and hepatic fat.

## Results

In this manuscript, we have conducted three inter-related studies: an observational study with two cohorts (Fenland and National Survey of Health and Development [NSHD] cohorts), two murine experiments involving genetic and dietary-induced hepatic steatosis, and a carbohydrate-overfeeding trial in humans. We designed the cohort analyses to identify key TAG species associated with hepatic fat in healthy adults; two murine experiments to determine whether the specific TAG species correlated with hepatic DNL and a ‘western diet’ (WD) high in both saturated fat and free sugar; and a human feeding intervention to test whether dietary carbohydrates influence the specific TAGs. In addition, in the cohort analysis, we examined whether the specific TAG species were associated with selected dietary, genetic and lifestyle factors. Finally, we confirmed our TAG biomarkers for fatty liver in samples from patients with biopsy confirmed liver steatosis.

**Fig. 1:**
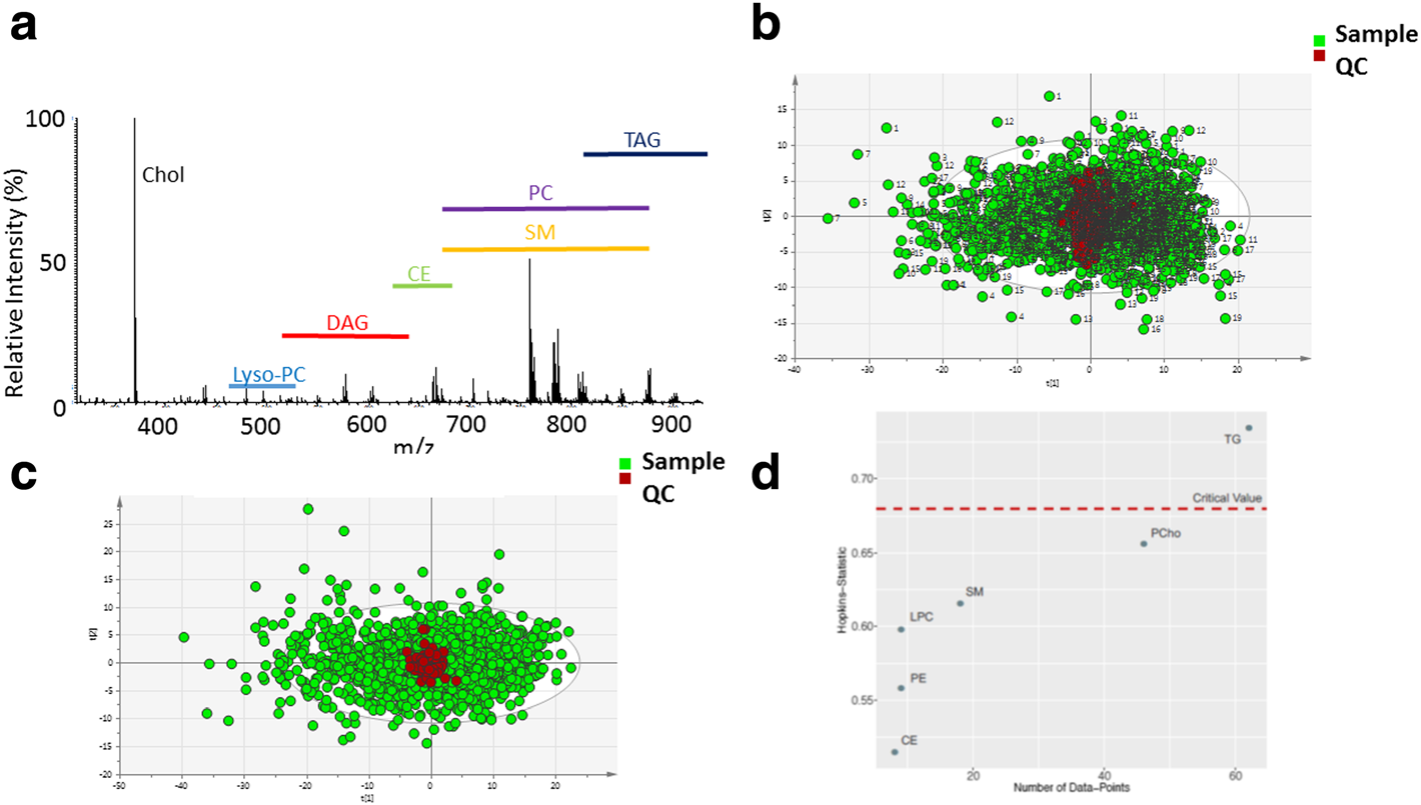
High-resolution direct infusion mass spectrometry (DI-MS) of blood plasma from the Fenland cohort. **a** High-resolution mass spectrum from a typical blood plasma sample analysed by DI-MS. chol cholesterol, lyso-PC lysophophatidylcholines, DAG diacylglycerols, CE cholesterol esters, PC phosphatidylcholines, SM sphingomyelins, TAG triacylglycerols **b** Principal component analysis (PCA) of samples from the Fenland cohort (*green circles*) and pooled quality control samples (*red circles*) before normalisation. **c** As (b) but following batch correction normalising the total intensity of QC samples across the 19 96-well-plates used to analyse the samples from Fenland. **d** Hopkin’s statistic of individual lipid classes reveals that only TAGs have significant substructure in the Fenland dataset. The critical value is displayed as a *dashed red line*. CE cholesterol esters. PE phosphatidylethanolamines, LPC lysophosphatidylcholines, SM sphingolipids, PC phosphatidylcholines, TG triacylglcerols

DI-MS of 1507 samples of the subset of the Fenland cohort detected 170 formula-assigned lipids common across the dataset, with the detection of lyso-phosphatidylcholines, diacylglycerols, phosphatidylcholines, phosphatidylethanolamines, sphingomyelins and TAGs (Fig. 1a). Before normalisation, the mean coefficient of variation (CV) for individual lipid species in the 100% QC pool sample across the study was 17%, with the QC samples clustering at the centre of the sub-cohort of Fenland when examined using principal component analysis (PCA) (Fig. 1b). Applying a simple normalisation whereby the total intensities of samples were corrected by normalising the quality control (QC) samples reduced the mean CV for all individual lipid species to 11%, further improving clustering of QC samples (Fig. 1c).

Next, to investigate clustering within the dataset, clustering tendency was tested using the Hopkin’s statistic [20] which indicated that the data points for individual lipids were non-uniformly distributed (H = 0.76, *p* < 0.05, where H → 1 indicates highly clustered, H = 0.5 randomly distributed and H = 0 uniformly distributed). Both hierarchical clustering and PCA indicated that TAGs and DAGs formed a distinct cluster from the other lipid species (Additional file 1: Figure S1a and S1b, Table S1). However, it should be noted that the Hopkin’s statistic is sensitive to the number of species in a given group and so may have missed clustering in the other lipid classes which have smaller numbers of lipid species associated with them. Next, the substructure of the non-glycerolipid species were investigated using PCA. This indicated that while phosphatidylcholines were relatively disperse, other species clustered according to lipid class (Additional file 1, Figure S1c and S1d), with similar results produced for K-means and self-organising maps (SOM) clustering (Additional file 1, Figure S1e and S1f). Hopkin’s statistics of each lipid class determined that only TAGs had statistically significant clustering tendency (Fig. 1d).

### DI-MS reveals discrete clusters of TAGs in human blood plasma

**Fig. 2:**
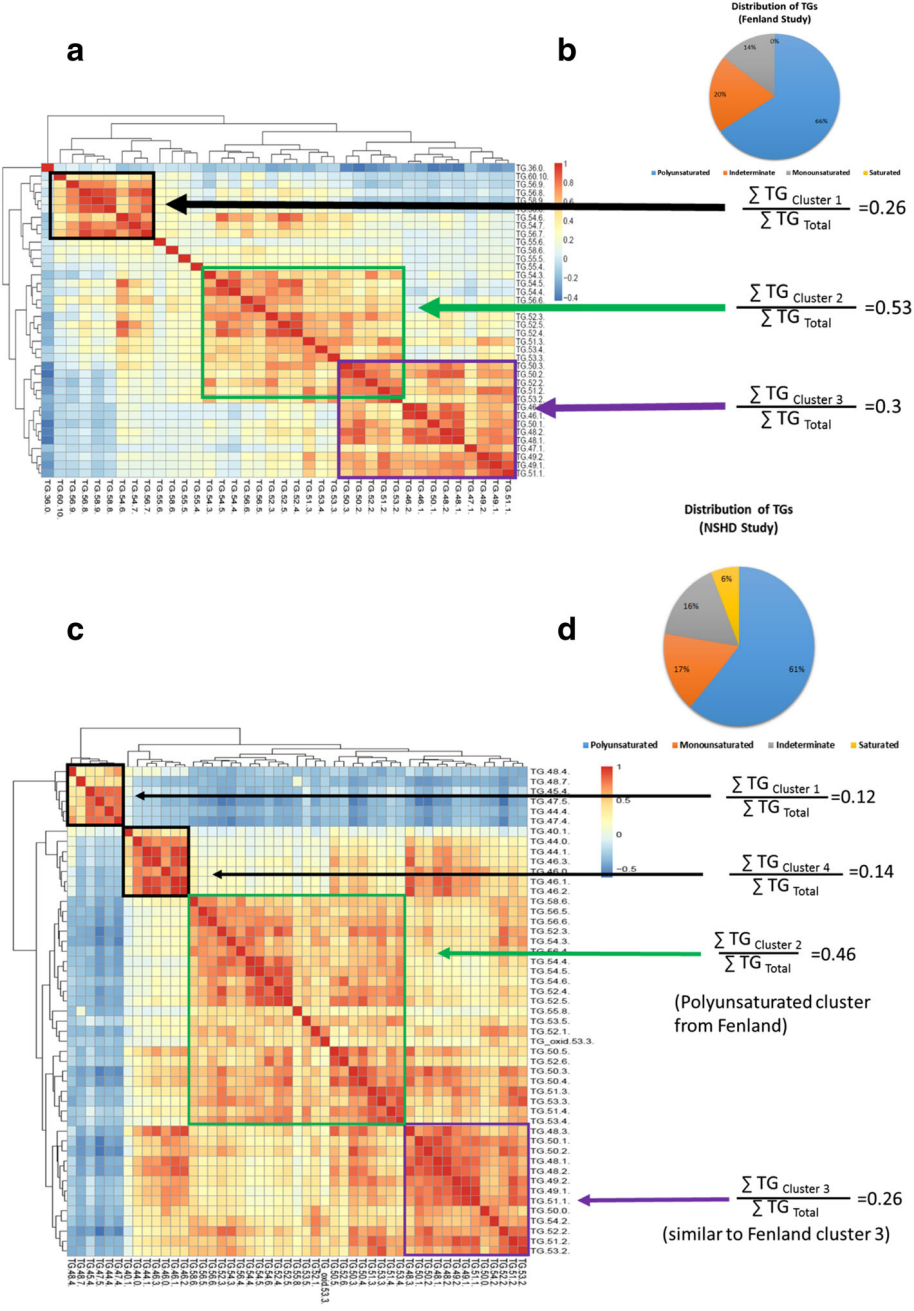
Examining the distribution of triacylglycerols (TAGs) in Fenland and National Survey of Health and Development (NSHD) cohorts. **a** Bayesian Hierarchical Cluster Analysis (B-HCA) of the TAG distributions as measured by DI-MS in the Fenland cohort. Relative intensities are shown of the major three clusters. TAGs are identified by total number of carbons in the fatty acid side chains: total number of double bonds in the fatty acid side chains. **b** *Pie chart* of the relative distribution of different TAGs in the Fenland cohort. **c** B-HCA of the TAG distributions as measured by DI-MS in the NSHD cohort. Relative intensities are shown of the major 3/4 clusters. **d** *Pie chart* of the relative distribution of different TAGs in the NSHD cohort

The TAG profiles were analysed by Bayesian Hierarchical clustering, producing three distinct clusters (Fig. 2a). This included a large cluster of TAGs containing shorter, more saturated FAs (cluster 3), as well as two large clusters containing more unsaturated FAs (clusters 1 and 2) (Fig. 2b). Using the integral values for each m/z for these TAG species it was possible to compare the relative intensities of these clusters, with cluster 3 representing ~ 30% of the TAG total intensities for the mass spectra.

### Replication of TAG profile in the NSHD cohort

To examine whether this clustering is present in other populations we examined the NSHD study, another UK cohort, and profiled the lipids within blood plasma from 1701 individuals. Again, Hopkin’s statistic indicated that sub-clustering was only present in the TAG species (Additional File 1, Figure S2). Focussing on the TAG species, again Bayesian hierarchical clustering produced distinct clusters of TAG species (Fig. 2c). Clusters 1 and 4 were dominated by TAGs containing very short, unsaturated FAs (cluster 1 ~ 12%) and short saturated/monounsaturated FAs (cluster 4 ~ 14%), respectively. Cluster 2 consisted of TAGs (~ 46%) containing longer, polyunsaturated FAs (~ 46%) similar in composition to those in cluster 2 of the Fenland cohort. Cluster 3 contained the TAGs found in cluster 3 of the Fenland cohort, containing saturated and monounsaturated FAs with ~ 16–18 carbons in length. There was also a similar distribution of TAGs compared to Fenland (Fig. 2d), although NSHD had a greater proportion of TAGs only containing saturated FAs. While more TAGs were detected in samples from NSHD, limiting comparisons to the 25 common TAGs between the two studies, overall there was similarity between the clusters identified in both cohorts (Additional File 1, Figure S3).

### TAG profile of adults with hepatic steatosis determined by the fatty liver index (Fenland)

**Fig. 3:**
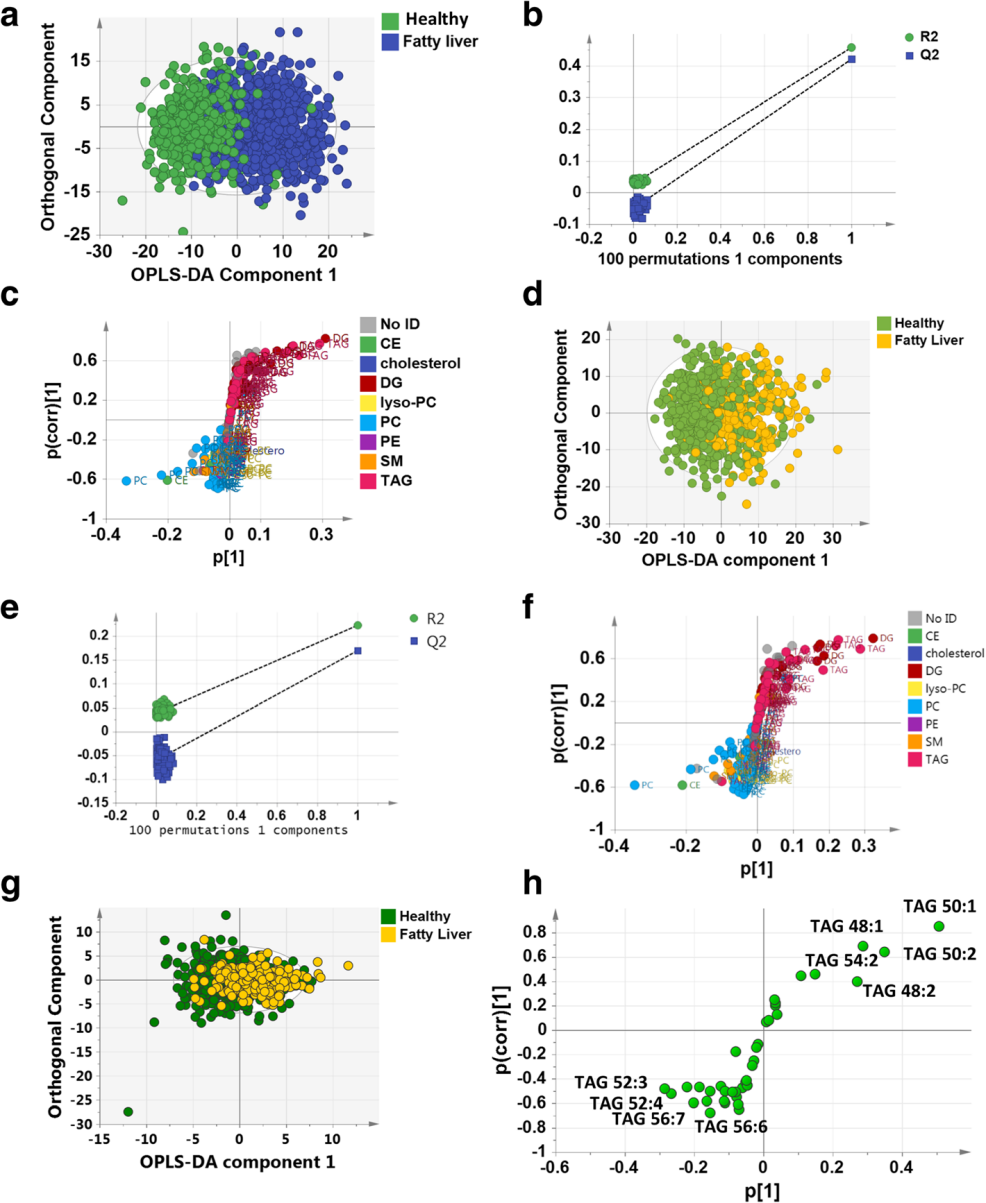
**a** Orthogonal partial least squares discriminant analysis (OPLS-DA) of individuals with (*blue*) and without (*green*) hepatic steatosis as assessed by fatty liver index. **b** Permutation validation of the plot in (**a**). R^2^ and Q^2^ values to the *left* of the plot are from random models compared with the values for the true model on the *right*. **c** S-plot of discriminatory metabolites in (**a**). Metabolites are coloured according to lipid class. **d** OPLS-DA of individuals with (*yellow*) and without (*green*) hepatic steatosis as assessed by ultrasound. **e** Permutation validation of the plot in (**d**). R^2^ and Q^2^ values to the *left* of the plot are from random models compared with the values for the true model on the *right*. **f** S-plot of discriminatory metabolites in (**d**). Metabolites are coloured according to lipid class. CE cholesterol ester, DG diacylglycerol, lyso-PC lyso-phosphatidylcholine, PC phosphatidylcholine, PE phosphatidylethanolamine, TAG triacylglycerol, SM sphingomyelin. **g** OPLS-DA scores plot of the TAG profile of individuals with (*yellow*) and without (*green*) hepatic steatosis as assessed by ultrasound. **h** S-plot of discriminatory TAGs in (**g**)

Fatty liver index (FLI) is a surrogate marker of fatty liver determined by ultrasound, composed of BMI, waist circumference, TAGs and gamma-glutamyl transferase (GGT) concentration [21, 22]. By this measure, steatosis was indicated in 493 participants (33%, FLI ≥ 60). A robust model could be built using orthogonal partial least squares discriminant analysis (OPLS-DA) to separate those with and without fatty liver disease according to the FLI score (the amount of variance in the data represented by the model (R^2^(X)) = 62%, R^2^(Y) = 46%, goodness of fit of the model (Q^2^) = 42%; Fig. 3a). The OPLS-DA model passed the random permutation test (Fig. 3b) and cross-validation analysis of variance (CV-ANOVA) (*p* < 1*10^−30^). This was driven by relative increases in TAGs and DAGs in the blood plasma of those with fatty liver disease, particularly those containing saturated and monounsaturated FAs ~ 16–18 carbons in length, alongside a relative decrease in phosphatidylcholines and cholesterol esters (Fig. 3c).

### TAG profile of adults with hepatic steatosis determined by ultrasound (Fenland)

As FLI is in part determined by serum TAG concentrations, it is possible that the association between FLI and the blood lipidome was driven by total TAG concentration and not hepatic steatosis, per se. To examine the association between the presence of hepatic steatosis and the plasma lipidome further, we examined the subset of 896 individuals who had been characterised by ultrasound and had good quality mass spectra in the sub-cohort of Fenland. This group consisted of 26% with mild-moderate steatosis (scores 5–10) and 74% with no steatosis (scores 3–4) (Table 1). OPLS-DA readily separated the two groups (R^2^(X) = 62%, R^2^Y = 22%, Q^2^ = 17%; Fig. 3d), and the model passed cross-validation by random permutation (Fig. 3e) and by CV-ANOVA (*p* = 5*10^−30^). Examining the variables responsible for this separation using an S-plot, blood plasma from individuals with fatty liver were most associated with increases in TAGs and DAGs, in particular those containing saturated and monounsaturated FAs with ~ 16–18 carbons in length, and relative decreases in phosphatidylcholines and cholesterol esters (Fig. 3f). To demonstrate the predictive capability of the model we created an OPLS-DA model of those with only FLI measures (train dataset) to predict the status of those with both FLI and ultrasound, producing a receiver operator curve (ROC) with an area under the curve (AUC) of 0.87 for the test dataset (Additional File 1: Figure S4a–d).

**Table 1:** Comparison of key anthropometric data for those with and without hepatic steatosis as measured by ultrasound in the Fenland cohort. A Student’s t-test was used to compare parameters between the two groups

	Non-steatosis	Steatosis
Individuals assessed by ultrasound (n)	663	233
Women (n (%))	424 (64.0)	110 (47.2)
Age (years)	45 ± 7	48 ± 7***
BMI (kg/m^2^)	25.5 ± 3.7	30.7 ± 4.8***
Fat mass (kg)	24.2 ± 7.5	33.8 ± 9.5***
Liver score	3.66 ± 0.47	6.06 ± 1.20***
Fasting plasma insulin (pmol/L)	37.9 ± 23.0	68.0 ± 49.4***
Fasting plasma NEFAs (μM)	355 ± 179	369 ± 181
Alcohol consumption (g/d)	9.4 ± 12.3	10.5 ± 14.1
Total carbohydrate consumption	243 ± 106	237 ± 89
Fasting blood glucose (mM)	4.78 ± 0.59	5.12 ± 0.74***
Fasting blood triglyceride (mM)	1.03 ± 0.65	1.70 ± 0.64***
HOMA-IR	0.80 ± 0.49	1.45 ± 1.04***
Fatty Liver Index	31.1 ± 25.4	70.0 ± 24.4***

***p < 0.001

To further examine the changes in TAGs associated with hepatic steatosis, we examined the TAG profiles for those volunteers who had had ultrasound measurements of fatty liver disease. As part of this analysis for each person the total intensity of all of the m/z peaks associated with TAGs was normalised to 100% and each individual TAG was represented as a percentage of this total. Again, OPLS-DA readily separated the two groups (R^2^(X) = 43%, R^2^Y = 11%, Q^2^ = 9%; Fig. 3g) and the model passed cross-validation by random permutation (Additional File 1, Figure S4e) and by CV-ANOVA (*p* = 8*10^−17^). Examining the variables responsible for this separation using an S-plot, blood plasma from individuals with fatty liver were most associated with increases in TAGs 54:2, 48:1, 48:2, 50:1 and 50:2 s and decreases in TAGs 52:3, 52:4, 56:7, 56:6, 54:4 and 56:8 (Fig. 3h).

### Murine studies of de novo lipogenesis

**Fig. 4:**
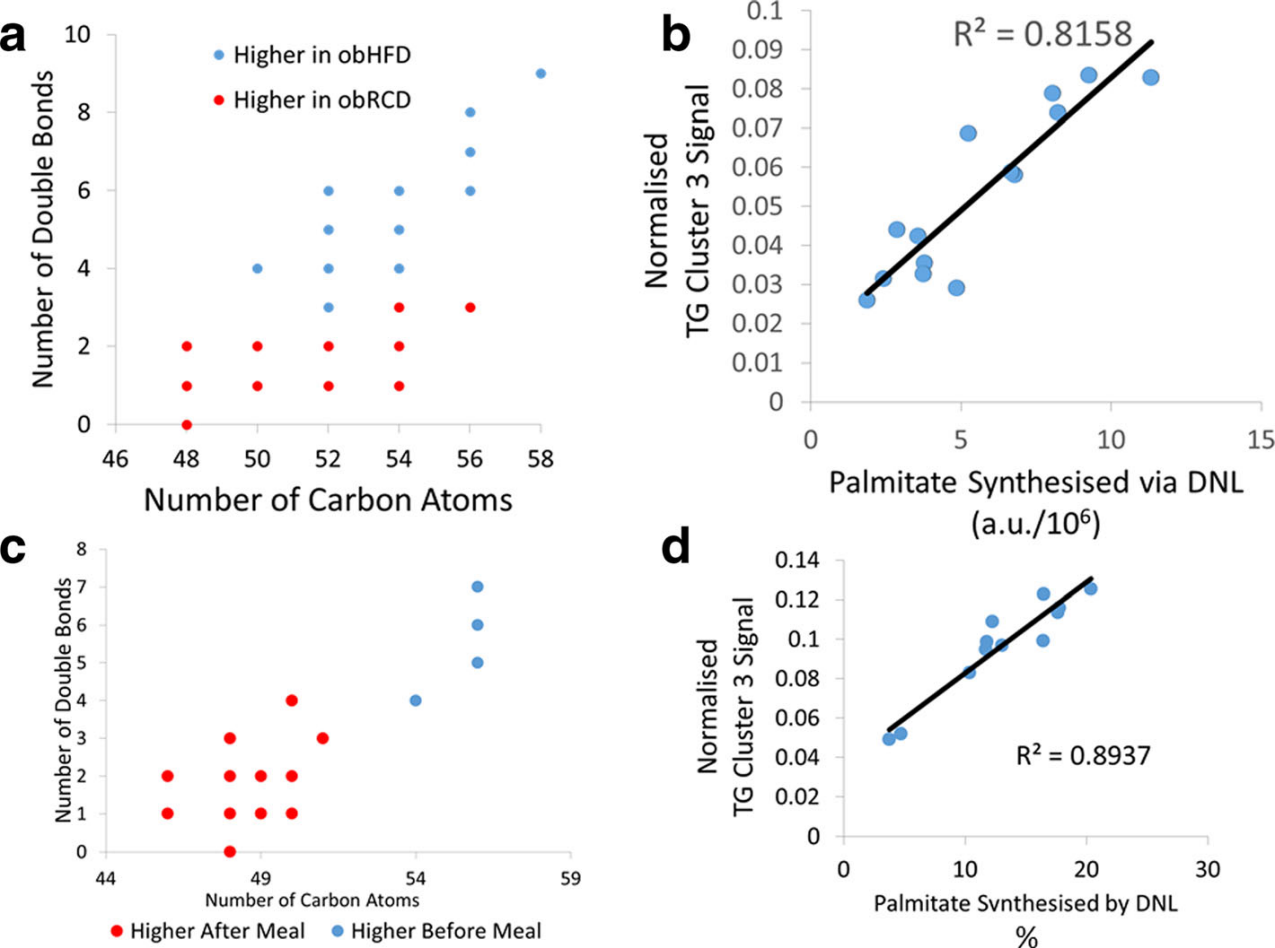
**a** Hepatic TAG changes associated with high fat or regular chow feeding in *ob*/*ob* mice. Using partial least squares discriminant analysis (PLS-DA) to assess the changes in the TAGs within the murine liver demonstrates a shift toward a higher proportion of TAGs with fewer double bonds and carbon atoms when fed regular chow than when fed a high fat diet. Each point’s area reflects the Variable Importance Parameter score for the PLS-DA model. **b** A simple linear model of the summated TAG Fenland cluster 3 signal against calculated de novo synthesised palmitate. Plotting the total normalised TAG cluster 3 signal against the total palmitate calculated to be synthesised from DNL using deuterium incorporation revealed a significant positive correlation between the two measurements (R^2^ = 0.82, *p* < 1*10^−5^). **c** Plasma TAG changes before and after a high carbohydrate meal. Using PLS-DA to assess the changes in the TAGs within the plasma lipidome demonstrates a shift toward a higher proportion of TAGs with fewer double bonds and carbon atoms 3.5 h after the high carbohydrate meal compared to before the meal in the fasting state. Each point’s area reflects the Variable Importance Parameter score for the PLS-DA model. **d** A simple linear model of the summated TAG Fenland cluster 3 signal against calculated de novo synthesised palmitate. Plotting the total normalised TAG cluster 3 signal against the proportion of palmitate calculated to be synthesised from DNL using deuterium incorporation revealed a significant positive correlation between the two measurements (R^2^ = 0.89, *p* < 5*10^−6^)

Male *ob/ob* and C57BL/6 control mice were fed either on regular chow diet (relatively high in carbohydrate) or on a high fat diet, and the rate of DNL followed by deuteration of palmitate from D_2_O enrichment in the mouse body water. Hepatic TAGs were analysed using LC-MS to assess any effect of diet or genotype on the TAG profile and whether these changes correlated with the rate of DNL. TAGs in *ob/ob* mice were compared between the dietary interventions using PLS-DA (R^2^X = 90%, R^2^Y = 99%, Q^2^ = 97%), which passed cross-validation by random permutation and CV-ANOVA (*p* = 5.85*10^−7^). O*b/ob* mice fed a regular chow diet had higher concentrations of TAGs with shorter, more saturated FA moieties compared with *ob/ob* mice on a high fat diet (Fig. 4a). A more complex pattern was detected for the wild-type (WT) strain, in part reflecting more modest DNL with an enrichment of TAGs with 50 and 52 carbons, but also reflecting the composition of the high fat diet (Additional File 1, Figure S5).

The synthesis of palmitate was directly measured by deuterium incorporation using GC-MS and isotope ratio mass spectrometry (IR-MS) to normalise for the body water deuterium enrichment in the *ob/ob* mice on a chow diet. The quantity of palmitate synthesis was assessed by linear regression for its correlation with the total peak intensity of each TAG detected in the mouse that relate back to clusters identified in the Fenland study (Fig. 4b). Fenland cluster 3 produced a positive correlation (R^2^ = 0.82, *p* < 1*10^−5^) while Fenland TAG cluster 2 showed no correlation with the rate of DNL in *ob/ob* mice (R^2^ = 0.001, *p* = 0.91) (Additional File 1, Figure S6). Fenland TAG cluster 1, however, demonstrated a negative correlation with rates of DNL, as assessed by the quantity of de novo synthesised palmitate (R^2^ = 0.70, *p* < 5*10^−4^) (Additional File 1, Figure S6). To examine these differences in DNL rate, we examined expression of acetyl CoA carboxylase, fatty acid synthase, stearoyl-CoA desaturase and elongase 6, confirming that HFD reduced expression of these transcripts in the respective mouse models (Additional File 1, Figure S7).

**Fig. 5:**
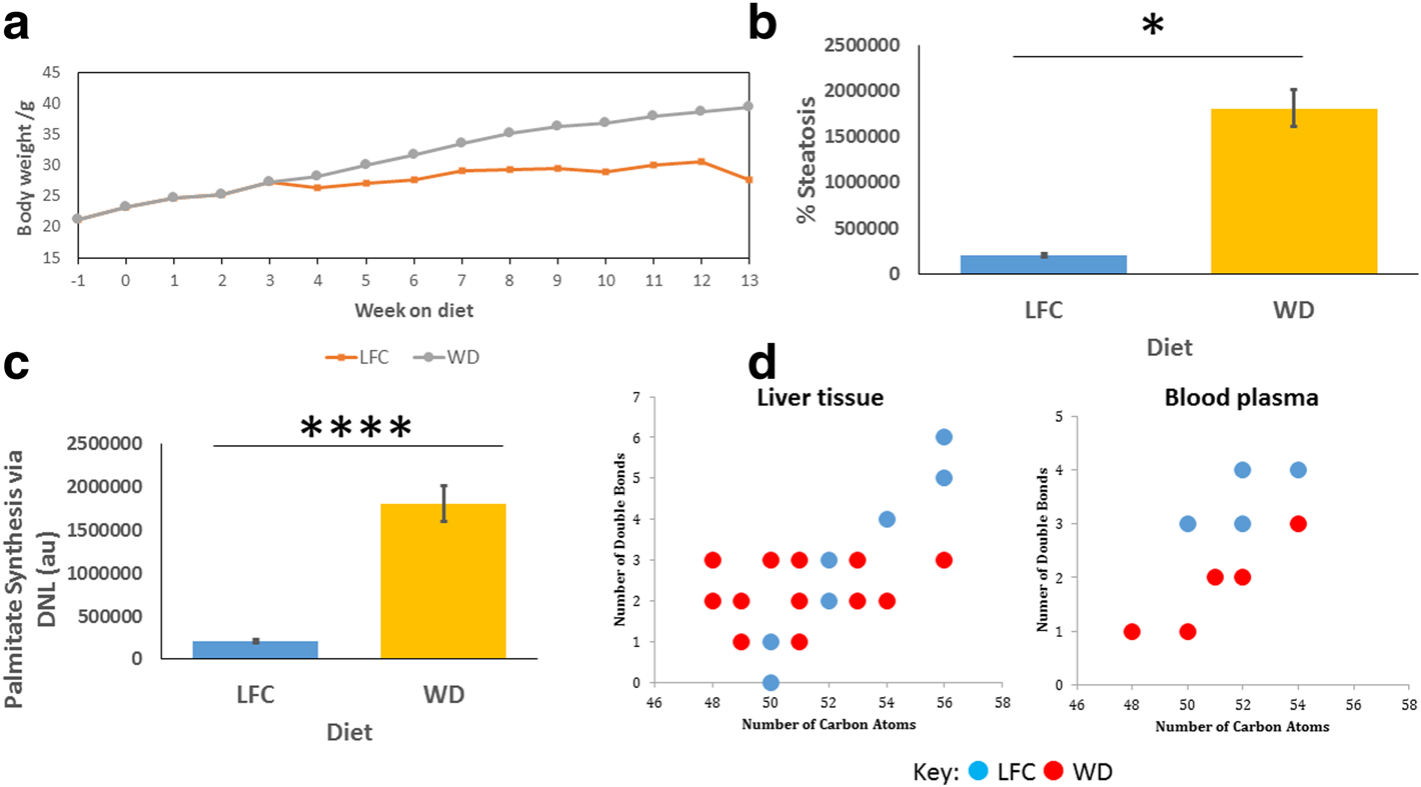
**a** Body mass of mice over the duration of the dietary intervention. WT mice fed low fat chow (LFC) were significantly lighter than those fed a Western diet (WD) from 6 weeks onward (*p* < 0.05; n = 8). b Histological assessment of hepatic steatosis. Masson’s trichrome stained sections were used for histological analysis and lipid content assessed as the average percentage of the hepatic tissue that appeared as unstained lipid droplets. Mice fed a LFC diet had significantly lower lipid content than mice fed WD. Mean ± SEM analysed by two-way ANOVA and Tukey’s multiple comparison test. **c** Overall quantity of DNL synthesised palmitate. Mice fed a WD demonstrate significantly increased production of palmitate compared to those fed LFC. Mean ± SEM analysed by two-way ANOVA and Tukey’s multiple comparison test. **d** LC-MS analysis of intact TAGs within the liver and blood plasma. TAGs that were significantly increased in each group were plotted by number of carbon atoms against number of double bonds within the FA moieties, when compared pairwise using PLS-DA with a VIP > 1 being taken as significant

Given that leptin has a profound effect on systemic metabolism, and that the *ob*/*ob* mouse does not represent just a model of hyperphagia but is also hypercorticosteronemic, we also studied the effects of a WD (high in both fat and free sugar) on NAFLD-associated promotion of DNL in WT mice. We fed WT mice either on a WD or low fat chow (LFC) diet, with the former diet inducing a significant weight gain from week 6 of feeding (*p* < 0.05; n = 8; Fig. 5a). Body composition of the mice measured using TD-NMR after five weeks and 12 weeks of the dietary intervention showed that while body composition stayed constant on the LFC, fat mass significantly increased on a WD from 21.0 ± 1.4% to 38.9 ± 1.2% (mean ± standard error; *p* < 0.0001, n = 8) although no differences were detected in intraperitoneal glucose tolerance tests at four and ten weeks of dietary intervention (data not shown). Histological sections of hepatic slices were stained using Masson’s trichrome and assessed for the percentage area of steatotic tissue. WT mice fed a WD demonstrated significantly increased steatosis compared to those fed LFC (*p* < 0.05, n = 8; Fig. 5b).

Palmitate was assessed for the total quantity produced via DNL using the incorporation of deuterium from D_2_O. WT mice fed a WD demonstrated a significantly increased quantity of DNL-synthesised palmitate compared with WT mice fed LFC (*p* < 0.0001, n = 8; Fig. 5c). LC-MS was used to measure the lipidome of liver tissue to investigate TAG changes induced by the two diets. Mice fed a WD demonstrated a significant alteration in TAG profile towards shorter chain, more saturated FA containing TAGs than LFC fed mice (R^2^ = 0.731, Q^2^ = 0.932, *p* = 8.02 × 10^−6^ for CV-ANOVA for the associated PLS-DA plot). Figure 5d displays the most changed TAGs in liver tissue between the two groups. Although mice appeared to have a more restricted number of TAGs within their blood sera than within the hepatic tissue, TAG profiles were compared using PLS-DA, revealing that mice fed a WD had an increased proportion of shorter chain more saturated TAGs than mice fed LFC (R^2^ = 0.519, Q^2^ = 0.877, *p* = 1.07 × 10^−4^ for CV-ANOVA for the associated PLS-DA plot). Figure 5d displays the most changed TAGs in blood sera between the two groups.

### Human carbohydrate feeding study in healthy volunteers

Plasma intact lipid profiles were examined following a high carbohydrate dietary intervention in ten healthy young adults using DI-MS. There was a significant change in the blood plasma TAG profile as detected by OPLS-DA (R^2^X = 56%, R^2^Y = 94%, Q^2^ = 86%), which passed cross-validation by random permutation and CV-ANOVA (*p* = 3.84*10^−6^). This model demonstrated an alteration towards shorter chain TAGs with fewer double bonds after a high carbohydrate meal compared with fasting, supporting the hypothesis that these TAGs are increased in concentration by DNL (Fig. 4c).

The rates of DNL, measured using deuterium incorporation into palmitate within the TAG lipid fraction, also increased after the meal, peaking at 3.5 h post morning meal. Across the linear increase in palmitate synthesised via DNL between 1 and 4 h after baseline measurements, the fractional synthetic rate of palmitate was 4.41 ± 1.73% hour^−1^. To assess the possible correlation between TAG cluster 3 in the Fenland study and the rate of DNL, the TAG cluster 3 signal was summated at each time point and analysed using simple linear modelling against the DNL rate at that time point (Fig. 4d). This resulted in a positive correlation between the measured rate of DNL at that time point and the sum of TAG cluster 3 peaks (R^2^ = 0.89, *p* < 5*10^−6^). Examining the second cluster of TAGs, containing longer, more unsaturated fatty acids and akin to clusters 1 and 2 in the Fenland cohort, there was a significant negative correlation with palmitate synthesised via DNL (Additional File 1**,** Figure S8).

### The association of other risk factors with the TAG cluster containing saturated FAs (Fenland)

To investigate further the TAGs associated with DNL, we performed a combination of linear regression and logistic regression of these factors with a range of other explanatory variables measured in the Fenland study (Additional File 1: Figure S9). The absolute level of each of the TAG species was increased with increasing hepatic steatosis. The series of multivariable adjusted analyses showed that lifestyle (diet and physical activity) and genetic factors (single nucleotide polymorphisms (SNP) previously associated with hepatic steatosis: SNPs LYPLAL1 (rs12137855); GCKR (rs780094); PPP1R3B (rs4240624); NCAN (rs2228603); PNPLA3 (rs738409); SREBP-1f (rs11868035)) had little impact on the associations (Additional File 1: Table S1). However, PPP1R3B (rs4240624)) was strongly associated with all the TAG markers and was unaffected by adjustment for age, sex or BMI (*p* < 0.02 for TAGs 46:1, 46:2, 48:1, 48:2 and 50:1) (Additional File 1: Table S2). HOMA-IR and visceral fat/waist circumference explained a high proportion of the association between individual TAGs and hepatic steatosis; the associations for TAG(46:1) and TAG(48:2) were most influenced by adjustment for HOMA-IR and TAG(48:1) and TAG(50:1) were most influenced by waist/visceral adiposity. However, when adjusting for these factors, a positive association remained between TAG (50:1), the most intense TAG species, and liver fat (*p* < 0.05), indicating this association remained after correcting for all covariates.

### Analysis of TAGs with FLI (Fenland)

Performing linear regression, TAG(48:1), TAG(48:2) and TAG(50:1) were associated with FLI after adjustment for all covariates (Additional File 1: Tables S3–6). In addition TAG(46:1) was associated with FLI adjusted for BMI but not after adjusting for waist or visceral adipose tissue volume. Thus, across both analyses the cluster of TAGs associated with shorter, more saturated FAs containing myristate, palmitate and stearate were associated with increased hepatic steatosis as measured by ultrasound and FLI. For TAG (48:1), TAG(48:2) and TAG (50:1), these associations remained after correction for all covariates including sex, age, genetic factors and lifestyle factors.

Dietary variables which have been associated with increased lipogenesis and liver fat include a WD rich in fat, particularly saturated fat, refined sugars and fructose (particularly in the context of beverages) [20, 23]. We investigated whether dietary variables were associated with TAGs to determine if dietary variables are mediators in the association between TAG markers and steatosis. The only dietary factors which appeared to modify the TAG markers were saturated fat and free sugar (WHO definition, analogous to non-milk extrinsic sugars-NMES). The association between saturated fat intake and these TAGs was independent of HOMA-IR, BMI and visceral fat (Additional File 1: Table S5). The association between proportion of free sugar in the habitual diet and most TAGs modelled was partly mediated by adiposity and insulin sensitivity (Additional File 1: Table S6).

### Specific TAGs are markers of liver fat in biopsy confirmed non-alcoholic steatohepatitis (NASH)

**Fig. 6:**
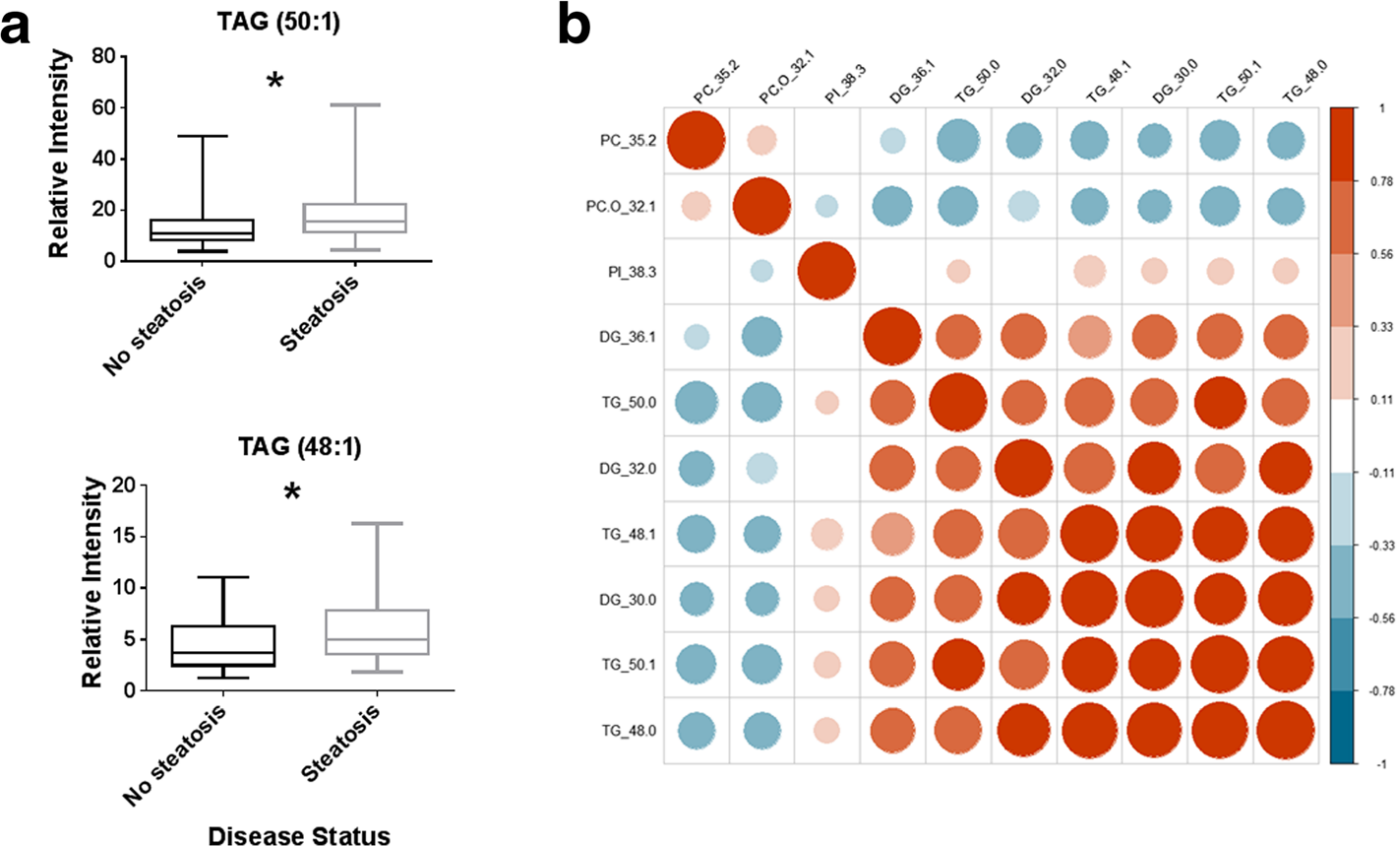
**a** *Boxplots* of the relative intensities of triacylglycerols TAG(50:1) and TAG(48:1) in blood plasma of patients with biopsy confirmed NASH separated according to ‘steatosis’ component of the NAFLD Activity score. No steatosis: score of 0 or 1, steatosis: score of 2 or 3. **b** *Heat map* of the correlation of the most discriminatory lipids between those with a low steatosis score (0 or 1) or high steatosis score (2 or 3). TG triacylglycerol, PC phosphatidylcholine, PI phosphatidylinositol, DG diacylglycerol

Finally, we confirmed the importance of the TAGs previously described in steatosis in the sera of a cohort of biopsy proven NASH patients (Table 2). When using the ‘steatosis’ component of the NAFLD activity score (NAS) to discriminate the amount of fat accumulation in the liver (0–1 vs 2–3), the lipid profiles of these patients showed TAG(50:0), TAG(48:1), TAG(50:1) and TAG(48:0) to be increased in the patients with higher fat accumulation (*p* = 0.043, 0.039, 0.034 and 0.0056, respectively; Student’s t-test) (Fig. 6a). These TAG species were found to be highly correlated across this patient group, in part confirming previous clusterings detected in the Fenland and NSHD cohorts (Fig. 6b).

**Table 2:** Characteristics of cohort of biopsy-proven NASH patients

	Steatosis 0–1	Steatosis 2–3	*p* (T-test)
n	40	36	/
M/F	22/18	24/12	NS ^*(Chi-Squared)*^
Age (years)	58.5 ± 1.7	53.6 ± 1.9	NS (0.06)
BMI (kg/m^2^)	31.6 ± 0.6	32.6 ± 0.9	NS
Metabolic assessment			
Glucose (mmol/L)	6.2 ± 0.3	7.0 ± 0.4	NS (0.1)
Insulin (pmol/L)	109.6 ± 11.0	130.7 ± 12.6	NS
HOMA2-IR	2.1 ± 0.2	2.5 ± 0.2	NS
TAGs (mmol/L)	1.8 ± 0.2	1.9 ± 0.3	NS
Total cholesterol (mmol/L)	4.3 ± 0.2	4.3 ± 0.1	NS
LDL cholesterol (mmol/L)	2.6 ± 0.2	2.6 ± 0.1	NS
HDL cholesterol (mmol/L)	1.0 ± 0.1	1.0 ± 0.03	NS
Liver function tests			
AST (U/L)	41.5 ± 3.8	46.7 ± 3.9	NS
ALT (U/L)	53.02 ± 3.9	72.9 ± 6.3	< 0.01*
ALP (U/L)	118.4 ± 9.5	104.1 ± 6.7	NS
Histology			
NASH activity score	2.8 ± 0.2	4.8 ± 0.2	< 0.01*
- Steatosis	0.8 ± 0.02	2.3 ± 0.1	< 0.01*
- Ballooning	0.7 ± 0.1	1.0 ± 0.1	0.03*
- Inflammation	1.4 ± 0.1	1.5 ± 0.1	NS
Fibrosis	1.5 ± 0.2	1.8 ± 0.2	NS

* *p* < 0.05

## Discussion

In this series of inter-related studies, we profiled the lipid content of > 3000 individuals from two large-scale cohort studies, two murine dietary intervention studies and a physiological study of DNL in order to investigate why a sub-set of TAGs is associated with the development of T2DM, possibly through the prior development of hepatic steatosis [10–14]. Of all the lipid species measured in our lipidomic assay, only TAGs showed evidence of sub-structure. Bayesian hierarchical cluster analysis revealed that there were three distinct clusters of TAGs that were found across all three human studies and also found to be repeated broadly in both WT and obese mice. We identified that one of these clusters, representing ~ 34% of the total TAG signal in the mass spectra, was selectively associated with hepatic steatosis, both as measured by ultrasound and FLI. To investigate this cluster of TAGs further, we performed three dietary/physiological interventions in mice and healthy humans to see whether increased DNL in turn could contribute to increases in these TAGs which contain shorter and more saturated FAs (FAs with 16–18 carbons which are saturated or monounsaturated). In the two experiments where DNL was also directly measured using GC-MS to detect the incorporation of deuterium into saturated FAs from D_2_O in the body, these TAGs were increased in concentration in high correlation with DNL. Interestingly, the contribution of TAGs containing longer, more unsaturated fatty acids were negatively correlated with DNL. Furthermore, these TAGs, with fatty acids containing 16-18 carbons which are saturated or monounsaturated,  were found to be increased in blood plasma from patients with biopsy confirmed hepatic steatosis.

We then investigated factors contributing to the plasma concentrations of these TAGs. We found that TAGs with 46–50 carbons with one or two double bonds were associated with hepatic steatosis even after adjusting for sex, age, genetic factors (SNPs known to be associated with fatty liver disease) and lifestyle factors (physical activity, dietary factors and alcohol). For TAGs 46:1 and 48:2, these lipids were associated with IR and hepatic steatosis, while TAGs 48:1 and 50:1 were associated with visceral fat and hepatic steatosis. Dietary factors, saturated fat and sugar intake were strongly associated with these TAGs, although this was strongest for relative proportion rather than total amount per se. Luukkonen et al. [24] also have reported increases in these TAGs in liver biopsies from individuals with high HOMA-IR in patients with NAFLD. One concern with using FLI as a proxy for hepatic steatosis is that this index uses total serum TAG as one of the measures to calculate the index. However, we used in addition both ultrasound and liver biopsy as independent approaches for measuring hepatic steatosis, giving comparable results to using FLI. This may suggest that the particular TAGs identified as being associated with hepatic steatosis may be more sensitive measures compared with total TAG content used in the FLI, especially as TAGs containing longer, more unsaturated fatty acids are negatively correlated with hepatic steatosis.

While DNL has classically been thought to play a relatively minor role in total saturated FA content of the mammalian body [8], Lambert et al. [16] identified a number of limitations to this generalisation. First, many of the early studies measuring DNL are in the fasting state, where DNL is suppressed [25]. Second, DNL rates are lower in healthy, lean individuals compared with obese and IR individuals [17, 26]. Moreover, Lambert et al. questioned whether the period of observation of labelling with deuterium from D_2_O was too short in previous studies as the appearance of lipid can be delayed significantly in intrahepatic stores. Indeed, they experimentally demonstrated that in individuals with high liver fat the contribution of DNL to VLDL TAG content doubled to ~ 23% compared with individuals with low liver fat. Increased DNL is associated with increased size of VLDL particles, with Fabbrini et al. [27] making the intriguing observation that while apoB100 production is proportional to VLDL secretion and DNL production of FAs in the liver in individuals with normal hepatic fat content, apoB100 production does not keep pace with TAG VLDL secretion in fatty liver disease, first leading to increased VLDL particle size and then increased hepatic TAG deposition as export of de novo FAs is impaired.

The analyses of TAGs containing 46–50 carbons with one or two double bonds identified two TAGs (48:1 and 50:1) associated with both visceral fat and liver fat and two others (46:1 and 48:2) associated with whole-body IR and liver fat. Whole-body IR has been identified as one of the major influences on hepatic steatosis [28]. Furthermore, it can be difficult to distinguish the contribution of DNL production of FAs in the liver and in adipose tissue in terms of the total blood plasma content. However, in a previous study of lipodystrophy we demonstrated that raised blood plasma concentrations of TAGs with 46–50 carbons with one or two double bonds were enriched in the VLDL fraction, strongly suggesting their hepatic origin [19]. In addition, Fabbrini et al. [29] examining obese individuals matched for either visceral adipose tissue (VAT) or intrahepatic TAG (IHTG) content provided compelling evidence that IHTG was a better predictor of hepatic, adipose and skeletal muscle insulin sensitivity and VLDL-TAG secretion than VAT.

The recent increase in prevalence of NAFLD [30], even in children [31], raises the question as to whether environmental factors and, in particular, diet may play an important role. In most western countries the nutritional guidance for the past 30–40 years has been to reduce total fat and cholesterol content in our diets. However, that has been at the same time as an increase in carbohydrate consumption, particularly in the form of sugary drinks and fruit juices which can rapidly raise the concentration of glucose and fructose in the blood, stimulating DNL [32]. Indeed, recent findings from the EPIC InterAct study of eight countries across Europe reported that the saturated FAs myristate, palmitate and stearate were associated with increased incidence of T2DM [15]. In that study, these saturated fatty acids were correlated with the consumption of sugary drinks, alcohol (another DNL substrate) and potatoes, suggesting that such carbohydrate rich foods are likely to stimulate DNL which in turn may contribute to increased circulating concentrations of these fatty acids [15]. However, it should be noted that in the Fenland and NSHD cohorts, the TAGs we detected containing shorter, more saturated fatty acids could also arise from food substances high in saturated fatty acids as well as from carbohydrates via DNL.

There has been much interest in the field of metabolomics in the generation of predictive markers of T2DM and its associated metabolic diseases [33, 34]. One of the most reproducible set of prospective biomarkers of IR and T2DM are TAGs with shorter, more saturated FAs, first reported in the Framingham cohort [10] and since reproduced in a number of large cohort studies as well as the investigation of rarer patient groups of T2DM [11, 12, 14, 19, 35]. In a cohort of 679 well-characterised individuals, where fatty liver was assessed either by magnetic resonance imaging or liver biopsy, a ‘lipid triplet’ biomarker based on TAG(16:0/18:0/18:1), PC(18:1/22:6) and PC(O-24:1/20:4) had a sensitivity of 69.1% and specificity of 73.8%, demonstrating the potential of TAGs containing saturated fatty acids as diagnostic markers [36].

In addition to investigating the association between different TAG species and fatty liver disease we also examined whether SNPs known to be associated with fatty liver disease also were associated with changes in the concentration of TAGs that were most increased in fatty disease. The SNP rs4240624 in protein phosphatase 1, regulatory subunit 3B (PPP1RB) was strongly associated with the blood plasma relative concentrations of TAGs 46:1, 46:2, 48:1, 48:2 and 50:1. PPP1RB encodes the catalytic subunit of the serine/threonine phosphatase, protein phosphatase-1 and in turn the phosphatase regulates glycogen synthesis in liver and skeletal muscle. Variants in the gene, including those found at rs4240624, have been associated with susceptibility to fatty liver disease [37, 38]. However, this is the first time that genetic variants in this gene have been directly associated with changes in blood lipidomics. A reduced capacity to store glucose as glycogen would increase the relative proportion of glucose being converted to fatty acids as part of DNL, providing a plausible mechanistic explanation for the association between PPP1RB and TAGs containing saturated and monounsaturated fatty acids derived from DNL.

An important remaining question is whether the TAG species identified as associated with hepatic steatosis are causal for or a consequence of the development of NAFLD. TAGs are thought to be relatively unreactive compared with other lipid classes and it is unlikely that TAGs themselves are responsible for the development of IR and subsequent T2DM [39]. However, TAGs are synthesised via DAGs and these lipids have been shown to interfere with insulin signalling by activating protein kinase CƐ (PKCƐ) which leads to reduced insulin-stimulated insulin receptor substrate 2 (IRS-2) tyrosine phosphorylation [40, 41]. Indeed, increasing TAG synthesis by overexpressing diacylglycerol acyltransferase 2 (DGAT2) specifically in the liver of mice has been shown to increase the total liver content of DAGs as well as TAGs, increase the activity of PKCƐ and reduce the phosphorylation of IRS-2 [42]. In addition, palmitate and DAGs from DNL have been specifically associated with increased ER stress. Obesity is responsible for suppression of insulin signalling by hyperactivation of c-jun N-terminal kinase (JNK) through increased ER stress [43, 44]. Saturated fats, in particular palmitate and stearate, have been shown to be potent inducers of ER stress and apoptosis in hepatic cells [45]. It is not clear whether palmitate and stearate act directly to modulate the cellular membranes or induce the synthesis of other lipotoxic intermediates. Wei et al. demonstrated palmitate induced ER-stress in the absence of ceramide accumulation [45], while Chan et al. linked fructose-induced ER stress in the mouse liver to increased DNL and accumulation of hepatic DAGs [46].

## Conclusions

We have identified a cluster of TAG species which together are associated with hepatic steatosis. These TAGs are increased in concentration during raised DNL, as well as being associated with the proportion of free sugar in habitual diet and dietary saturated fat, and may provide a more sensitive marker for the metabolic consequences of fatty liver disease than measurements of total blood TAG concentrations.

## Methods

### Human cohort studies

*Fenland:* Plasma samples from 1507 individuals from a sub-set of the Fenland cohort (http://www.mrc-epid.cam.ac.uk/Research/Studies/Fenland/; Table 3) were stored at − 80 °C before analysis. The liver fat content of individuals was assessed by ultrasound for 901 participants. Analyses were compared with FLI, based on BMI, waist circumference, total TAGs and plasma levels of GGT [21]. Further details are given in the supplementary methods section.

**Table 3:** Comparison of the two cohorts used in the manuscript. Fenland and the National Survey of Health and Development (NSHD) are UK prospective cohort studies. Fenland (http://www.mrc-epid.cam.ac.uk/research/studies/fenland/) investigates the interaction between environmental and genetic factors in determining obesity, type 2 diabetes and related metabolic disorders. NSHD (http://www.nshd.mrc.ac.uk) is a British birth cohort study investigating the social and biological factors that affect lifelong health, ageing and the development of chronic disease risk

	Analysis of the sub-cohort of Fenland	NSHD
Individuals (n)	1507	1701
Hepatic steatosis assessed by ultrasound (n)	901	Not determined
Hepatic steatosis assessed by Fatty Liver Index (n)	1507	Not determined
Women (%)	55.8	52.1
Age (years)	45 ± 7	62 ± 2
Fasting blood triglyceride (mM)	1.22 ± 0.84	1.08 ± 0.10
BMI (kg/m^2^)	27.0 ± 4.8	27.6 ± 4.5

*Medical Research Council NSHD cohort*: To replicate the TAG identification in the Fenland cohort, plasma samples from 1701 individuals were analysed from the NSHD cohort [47]. Summary statistics for this cohort are presented in Table 3. Fatty liver status was not assessed in this cohort.

### Murine dietary intervention studies

All procedures involving mice were performed by a licence holder in accordance with the UK Home Office Animals (Scientific Procedures) Act 1986, and the University of Cambridge Animal Welfare and Ethical Review Committee.

In the first study, six-week-old male *ob/ob* mice and C57BL/6 controls were fed on regular chow diet (energy composition: 11.5% fat, 26.9% protein, 61.6% carbohydrate; Rat & Mouse No. I Maintenance; Special Diet Services, UK) or a high-fat diet previously described [48] (energy composition: 55% fat, 29% protein, and 16% carbohydrate; diet code: 829197; Special Diet Services, UK) (n = 7 per diet and genotype). The FA composition of the high fat diet was 25% polyunsaturated FAs, 48% monounsaturated FAs and 27% saturated FAs. Temperature was maintained at 20 ± 4 °C with a 12 h light/dark cycle. All mice drank water enriched with deuterium adjusted for each group depending on diet and average mouse weight to gain roughly 1% enrichment in body water for analysis of DNL. This was maintained for 14 days to achieve steady-state of enrichment. Animals were killed by CO_2_ asphyxiation, exsanguinated and dissected to collect hepatic tissue, which was flash frozen in liquid nitrogen and stored.

In the second study, five-week-old male C57bBl/6 mice (n = 8) were fed either a LFC diet (TD08485, Teklad Custom Research Diets, Envigo, Huntingdon, UK) or WD (TD88137) for 12 weeks. At the end of the fourth and tenth week each mouse underwent an intraperitoneal glucose tolerance test (IP GTT), and during the fifth and 12th week of the dietary intervention, the body composition of the mice was analysed by time domain nuclear magnetic resonance (TD-NMR) using a Bruker LF90 Minispec (Bruker Corp., Coventry, UK). After the second IP GTT mice were provided with D_2_O-enriched drinking water adjusted for each group depending on diet and average mouse weight to gain roughly 1% enrichment in the mice’s body water for the analysis of DNL. This was maintained for the final two weeks of the study to allow sufficient steady-state enrichment of the body water. Twelve weeks after the start of the dietary intervention mice were killed by cervical dislocation. The animals were then exsanguinated and dissected to collect hepatic tissue, which was flash frozen in liquid nitrogen and stored at − 80 °C until required for analysis. Hepatic samples for each mouse were also fixed in formalin for histological analysis. Serum was isolated from the collected blood by centrifugation at 3000 g for 10 min and stored at − 80 °C until analysis.

### Carbohydrate overfeeding in healthy human adults

This study was approved by Cambridge South Research Ethics Service Committee (Ref. no. 14/EE/0054). After an overnight fast, ten healthy volunteers (BMI = 22.2 ± 1.2 kg/m^2^, fasting blood triglyceride = 1.0 ± 0.5 mM, fasting blood glucose = 4.9 ± 0.4 mM) were provided with breakfast comprising 45% of their basal metabolic rate (BMR) [49] (61.5% carbohydrate [CHO], 21.2% fat and 17.3% protein by energy composition). Deuterium-labelled water (3 g/kg body water) was provided in two portions of equal sizes. After 3.5 h they were provided with a meal, which contained 47.5% of their BMR (65.4% CHO, 22.6% fat and 12.0% protein). The evening meal contained 47.5% BMR (61.1% CHO, 23.9% fat and 15.0% protein). The following morning a basal blood sample was taken for fasted state isotopic compositions of lipids and lipid profiling. A breakfast, identical to the previous morning, was provided, and blood samples (12 mL per sample) were taken every hour for 4 h. Lunch contained energy equivalent to 110% of BMR (69.7% CHO, 22.1% fat and 8.2% protein). Hourly blood samples (12 mL) were taken for a further 5 h.

#### Mass spectrometry

##### DI-MS of blood plasma in the cohorts and feeding studies

The method has been described previously [19] and is given in detail in the supplementary information. Lipid species from 15 μL of blood plasma were analysed on an Advion Triversa Nanomate interfaced with an Exactive MS (Thermo Scientific, Hemel Hempstead, UK). Features of interest were subsequently confirmed using fragmentation experiments on a Thermo Velos Orbitrap Elite MS in conjunction with chromatography using the ultra-high performance liquid chromatography (UHPLC) U3000 unit.

##### LC-MS of mouse liver tissue

LC-MS experiments were carried out using Thermo Scientific U3000 Autosampler coupled to a Thermo Scientific Elite™ Iontrap-Orbitrap Hybrid MS (Thermo Scientific, Hemel Hempstead, Hertfordshire, UK). A total of 5 μL of extracted tissue was injected onto an Acuity C18 BEH column (Waters™ T3 50 × 2.1 mm, 1.7 μm) maintained at 55 °C. Further details are given in the supplementary data.

##### GC-MS of mouse liver tissue to determine relative enrichment of TAG FAs following DNL

Lipids were pre-fractionated using solid phase extraction (SPE) with 50 mg sodium sulphate/NH_2_ 100 mg cartridges (Agilent Technologies, Santa Clara, CA, USA). The fractional synthetic rate of TAG FAs was determined using the incorporation of deuterium into TAG FA methyl esters (FAMEs) from deuterium-enriched water as previously described [17, 50].

##### Statistical analysis

*The Fenland cohort:* We tested associations of TAG(46:1); TAG(46:2); TAG(48:1); TAG(48:2) and TAG(50:1) with hepatic steatosis as assessed by ultrasound and FLI using ordinal logistic regression and estimating odds ratios. All analyses were adjusted for analysis batch and total TAGs. The analyses were further adjusted for potential confounders and mediators that could influence TAGs and hepatic steatosis including intrinsic characteristics (age, sex, genotype [SNPs LYPLAL1 (rs12137855); GCKR (rs780094); PPP1R3B (rs4240624); NCAN (rs2228603); PNPLA3 (rs738409); SREBP-1f (rs11868035)]) and lifestyle characteristics (dietary variables, physical activity, smoking, alcohol intake). We further evaluated whether the associations of TAGs with hepatic steatosis were explained by metabolic perturbations (insulin sensitivity, obesity and central adiposity). All cohort analyses were conducted using Stata version 14 (StataCorp LP, TX, USA). Further details are provided in the supplementary methods.

*Multivariate analyses of the cohorts, mouse study and human carbohydrate overfeeding study*: For multivariate analysis, all data were mean centred and Pareto scaled before subsequent analysis. Clustering tendencies of data were visualised using the package ‘pheatmap’, and Hopkin’s statistics, *H*, were calculated using the package ‘factoextra’ within R. The null hypothesis that the data has no substructure is rejected at the 95% confidence level if *H* > 0.6799 [51]. For further exploration of clustering within the whole lipidomic datasets agglomerative hierarchical clustering (from the ‘stats’ package) with‘Complete’ (farthest neighbour) linkage and Euclidean distance [52], the k-means algorithm (from the ‘stats’ package) was run with a Euclidean distance measure, and the self-organising maps method, from the ‘SOMbrero’ package in R [53] all within R. To further explore the clustering within the TAG species, Bayesian hierarchical cluster analysis as described by Heller and Ghahramani [54] was performed within R to compare the profiles of TAG species across the Fenland and NSHD datasets. PCA and OPLS-DA was used within Simca (version 13, Umetrics, Umea, Sweden) to compare lipid profiles. Model validity was assessed by R^2^, Q^2^, the random permutation test and CV-ANOVA within the Simca package.

## Additional file

Additional file 1: contains all the additional supplementary data in the form of a Word document. (DOCX 1130 kb)

## Abbreviations

CV: Coefficient of variation
CV-ANOVA: Cross-validation analysis of variance
CVD: Cardiovascular disease
DI-MS: Direct infusion mass spectrometry
DNL: De novo lipogenesis
FA: Fatty acid
FLI: Fatty liver index
GC-MS: Gas chromatography mass spectrometry
HOMA-IR: Homeostatic model assessment of insulin resistance
LC-MS: Liquid chromatography mass spectrometry
NAFLD: Non-alcoholic fatty liver disease
NAS: NAFLD activity score
NASH: Non-alcoholic steatohepatitis
NSHD: Medical Research Council National Survey of Health and Development
OPLS-DA: Orthogonal partial least squares discriminant analysis
PCA: Principal component analysis
QC: Quality control
T2DM: Type 2 diabetes mellitus
TAG: Triacylglycerol
