# Supplementary material for: Hepatic steatosis risk is partly driven by increased de novo lipogenesis following carbohydrate consumption
Source: Genome Biol. 2018 Jun 20;19:79. doi: 10.1186/s13059-018-1439-8 (PMC6009819; doi:10.1186/s13059-018-1439-8)
Supplement: Supplementary file 1 — contains all the additional supplementary data in the form of a Word document. (DOCX 1130 kb) [file 13059_2018_1439_MOESM1_ESM.docx]

**Supplementary data: A lipidomic population and translational feeding study of hepatic steatosis**

**Francis W.B. Sanders^1^, Animesh Acharjee^1, 2^*, Celia Walker^1*^, Luke Marney^1^, Lee D. Roberts^1,2,3^, Fumiaki Imamura^4^, Benjamin Jenkins^1^, Jack Case^1^, Sumantra Ray^1,5^, Samuel Virtue^6^, Antonio Vidal-Puig^6^, Diana Kuh^7^, Rebecca Hardy^7^, Michael Allison^8^, Nita Forouhi^4^, Andrew J. Murray^9^, Nick Wareham^4^, Michele Vacca^1, 2, 5, 6^, Albert Koulman^1^, & Julian L. Griffin^1,2#^**

1. MRC Human Nutrition Research, Fulbourn Road, Cambridge, UK. 2. Department of Biochemistry and the Cambridge Systems Biology Centre, University of Cambridge, Tennis Court Road, Cambridge, CB2 1GA, UK. 3. Current address: Leeds Institute of Cardiovascular and Metabolic Medicine, University of Leeds, Leeds, UK. 4. MRC Epidemiology, University of Cambridge, Addenbrooke’s Hospital, Cambridge, UK. 5. NNEdPro Global Centre for Nutrition and Health, Cambridge, UK. 6. Institute of Metabolic Science, University of Cambridge, Cambridge, UK. 7. MRC Unit for Lifelong Health and Ageing at UCL, London, UK. 8. Liver Unit, Department of Medicine, Cambridge Biomedical Research Centre, Cambridge University Hospitals NHS Foundation Trust, Cambridge, UK. 9. Department of Physiology, Development and Neuroscience, University of Cambridge, Cambridge, UK.

*contributed equally.

**Supplementary Methods:**

**Fenland methods**

*Cohort*

This study was designed to investigate the interaction between environment and genetic factors in determining intermediate quantitative traits related to obesity and type 2 diabetes, conducted in Cambridgeshire in the UK (http://www.mrc-epid.cam.ac.uk/research/studies/fenland-study/). Recruitment of the full cohort of participants born between 1950 and 1975 (aged 30-62 years at recruitment) began in 2005 through general practice lists in Ely, Wisbech, Cambridge, and surrounding villages as described previously [1]. Dietary data were collected by food frequency questionnaire [2]. Obesity was assessed by BMI and central adiposity by visceral adipose tissue volume assessed by Lunar Prodigy whole-body scanner (GE Medical Systems, Madison, WI). HOMA-IR was used as a marker of insulin resistance (<https://www.dtu.ox.ac.uk/homacalculator/>).

Full lipidomic analysis was conducted on the sub-sample of the first 1505 participants analysed here. Participants who self-reported to be taking lipid lowering medication (n=39) and participants who were non-plausible dietary reporters (n=79, see below) were excluded from analyses. The sample was used to explore whether palmitate and myristate containing triglycerides are markers of hepatic steatosis, and to determine whether this relationship is affected by the impact of genetic, lifestyle and metabolic factors related to fatty liver.

*Assessment of hepatic steatosis*

The liver fat content was assessed by ultrasound as described previously (ref). The score is assigned according to indications of normal (≤4), mild (5-7), moderate (8-10) or severe liver steatosis (≥11) based on increased echo reflectivity of the liver parenchyma (bright liver in comparison with kidney); decreased visualisation of the intra-hepatic vasculature; and attenuation of the ultrasound beam. Ultrasound data were available for 901 participants and 778 participants not on lipid medication and who were plausible dietary reporters (60% of the sub-cohort) and the liver score ranged from 3-10 with 26% of the 901 participants categorised to have mild-moderate steatosis (score 5-10) and 74% have no indication of steatosis (score 3-4).

Analyses were compared with fatty liver index (FLI) an algorithm based on BMI, waist circumference, triglycerides and gamma-glutamyl transferase (GGT) [3] which has been externally validated against MRS-confirmed fatty liver {Cuthbertson, 2014 #1011}. By this measure which ranges between 0-100, the median value was 37 (IQR = 57.5), steatosis was ruled out in 652 participants (43%, FLI <30) and was indicated in 493 participants (37%, FLI ≥60). In this cohort, sensitivity of detecting steatosis by FLI compared to ultrasound was 72% and specificity was 83%. FLI data were available for 1505 participants (1294 who were not on lipid medication, and for which there were plausible dietary values).

*SNP selection*

Loci which had previously been found to be associated with liver fat (assessed by computerised tomography and confirmed by biopsy) in a meta-analysis of genome-wide association studies[4] were explored for potential associations with the palmitate and myristate containing triglycerides. The loci explored were LYPLAL1 (rs12137855); GCKR (rs780094); PPP1R3B (rs4240624); NCAN (rs2228603); PNPLA3 (rs738409). A SNP (rs11868035) from the candidate gene SREBP-1f, which had previously been associated with non-alcoholic fatty liver disease (confirmed by biopsy) {Musso, 2013 #928} was also considered as a confounding factor or potential mediator of any association between liver fat and palmitate and myristate containing triglycerides.

*Dietary data*

Dietary data was collected by externally validated food frequency questionnaire (FFQ) and analysed using FETA 2.53 software (http://www.srl.cam.ac.uk/epic/epicffq/)[2]. Nutrient intake from all reported foods was calculated as average intake per day (g) and all data used for analysis were calculated as nutrients as a proportion of total energy. Implausible reporters were identified using the methods of McCrory *et al.* {Mccrory, 2002 #1013} following the principles of Black *et al.* {Black, 2000 #1014} using a cut-off of 2SD, which excluded 76 individuals (of 1407 dietary reports).

Dietary variables considered for analyses included those previously associated with increased lipogenesis and/ or liver fat and included a diet rich in fat, particularly saturated fat, refined sugars and fructose (although this was in the context of beverages) {Conlon, 2013 #959;de Wit, 2012 #960}. However, there is evidence that the impact of diet on liver fat is mediated through body fat {de Wit, 2012 #960;Green, 2014 #962}. Saturated fat, free sugar and fructose were all associated with the palmitate and myristate containing triglycerides and with liver score. Free sugar was defined using the WHO definition of all monosaccharides and disaccharides added to foods by the manufacturer, cook or consumer, plus sugars naturally present in honey, syrups and fruit juices {WHO/FAO, 2003 #904}. This was calculated using modified methods for calculating non-extrinsic milk sugars. Fructose and saturated fat were derived from all FFQ food consumed, but not beverages.

*Other lifestyle measures*

Smoking was assessed by questionnaire and categorised as never smoked; ex-smoker or current smoker. Alcohol intake was determined by questionnaire and converted into average g of absolute ethanol intake per week as a continuous variable. Physical activity energy expenditure was assessed by combined heart rate and accelerometer.

*Physiological measures*

Obesity was assessed by BMI and central adiposity by visceral adipose tissue volume assessed by Lunar Prodigy whole-body scanner (GE Medical Systems, Madison, WI) and reprocessed with iDEXA software (GE Medical Systems, Madison, WI). An indication of insulin resistance was assessed by HOMA2 (<https://www.dtu.ox.ac.uk/homacalculator/>).

*Statistical analysis*

In order to explore whether palmitate and myristate containing triglycerides are markers of hepatic steatosis we tested associations between the predictors TG46:1; TG 46:2; TG48:1; TG48:2 and TG50:1 (TG46:0 and TG48:0 were not detectible in this dataset) with the outcome liver score (assessed by ultrasound) using ordinal logistic regression. All analyses were adjusted for lipidomic analysis batch, total triglyceride, age and gender. The relationships were then adjusted for mediators and possible confounders built up according to our hypothetical model of factors impacting on hepatic steatosis (show theoretical model). We built up the model from the left (see figure): intrinsic characteristics (age, gender, SNPs), followed by lifestyle characteristics (potential dietary variables, physical activity, smoking, alcohol intake), followed by metabolic perturbations (insulin sensitivity, obesity and central adiposity). In order to explore which variables impacted on the relationship between the markers and liver fat, we retained all the variables while building up the models, regardless of whether they were significant.

Additionally the association between TG46:1; TG 46:2; TG48:1; TG48:2 and TG50:1 and FLI was assessed by linear regression analysis and using the same covariates and potential confounders as above.

Variable transformations were conducted on highly skewed data: TG46:1; TG48:1; TG48:2; TG50:1; alcohol intake, HOMA-IR, BMI, were all log (n) transformed, TG46:2 was log (10) transformed, visceral adipose tissue volume had a square root transformation.

In order to determine the direct relationship between palmitate and myristate containing triglycerides and liver fat, and the extent to which this relationship is mediated through abdominal adiposity, insulin resistance and other potential mediators the method of comparing coefficients of nested non-linear probability models (The “KHB” method) was used {Kohler, 2011 #966}. This is a general decomposition method, unaffected by rescaling or attenuation bias of non-linear models. The proportion of the relationship of each of the TG markers with liver score which is mediated through intrinsic, lifestyle, genetic and metabolic factors was assessed, controlling for total triglyceride, age and sex in the full and reduced models. All possible mediators explored in the initial analysis were included. The estimated difference between the total effect of the full model and the direct effect (between triglyceride markers and liver score) is the indirect or mediating effect. The relative contributions of all the mediators to the indirect effect was also calculated.

In order to assess the prognostic potential of palmitate and myristate containing triglycerides as a marker of liver fat, the area under the receiver operator curve (aROC) of these markers in addition to traditional indicators (age, sex, BMI and lipid medication ± ALT) was compared to the aROC of traditional markers alone.

All cohort analyses were conducted using Stata version 14 (StataCorp LP, TX, USA).

***Mass spectrometry measures of blood plasma***

The method has been described previously [5]. Lipid species from 15 μl of blood plasma were extracted with methyl tert-butyl ether. Samples were analysed on an Advion Triversa Nanomate interfaced with an Exactive MS (Thermo Scientific, Hemel Hempstead, UK). A mass acquisition window from 200 to 2000 m/z was used and acquisition was performed in positive mode. Automated compound annotation was carried out using both an exact mass search in compound libraries as well as applying the referenced Kendrick mass defect approach. Features of interest were subsequently confirmed using fragmentation experiments on a Thermo Velos Orbitrap Elite MS in conjunction with chromatography using the ultra-high performance liquid chromatography (UHPLC) U3000 unit.

*GC-MS of mouse liver tissue to determine relative enrichment of TAG FAs following DNL*

TAGs from the organic extracts of murine liver tissue (50 mg) or human blood plasma (100 μL) from the carbohydrate overfeeding study were separated by solid phase extraction, and derivatised using methanolic boron trifluoride. To this, 100 μL of chloroform: methanol (1:1) was added, followed by D_25_ tridecanoic acid in chloroform as an internal standard. The samples were heated at 80 **°**C for 90 min, then 300 μL of deionised water and 600 μL of hexane was added. The organic fraction was dried under nitrogen, and reconstituted in 200 μL of hexane. One μL of sample was injected onto an HP88 GC column (88% - cyanopropyl)aryl-polysiloxane, 0.17 μm film thickness, 320 μm diameter, 50 m length, Agilent Technologies, Santa Clara, USA) in a 6890 GC chromatogram coupled to a 5973 MS (Agilent Technologies (injector temperature = 250 °C, split ratio = 10:1, flow rate of helium = 12 mL min^-1^).

**Supplementary Results**

To investigate clustering within the dataset, clustering tendency was tested using the Hopkin’s statistic [6] which indicated that the data points for individual lipids were non-uniformly distributed (H=0.76, p<0.05). Both hierarchical clustering and PCA indicated that TAGs and DAGs formed a distinct cluster from the other lipid species (**Supplementary Figure 1a & 1b, Supplementary Table 1**). Next the substructure of the non-glycerolipid species were investigated using PCA. This indicated that while phosphatidylcholines were relative disperse, other species clustered according to lipid class (**Supplementary Figure 1c & 1d**), with similar results produced for K-means and SOM clustering (**Supplementary Figure 1e & 1f**). Hopkin’s statistics of each lipid class determined that only TAGs had statistically significant clustering tendency (**Figure 1d**) - this implies that all other co-regulated subgroups do not have an identifiable internal structure in this dataset. To examine whether this clustering is present in other populations we examined the NSHD study, another UK cohort, and profiled the lipids within blood plasma from 1701 individuals. Again, Hopkin’s statistic indicated that sub-clustering was only present in the TAG species (**Supplementary Figure 2**).

**
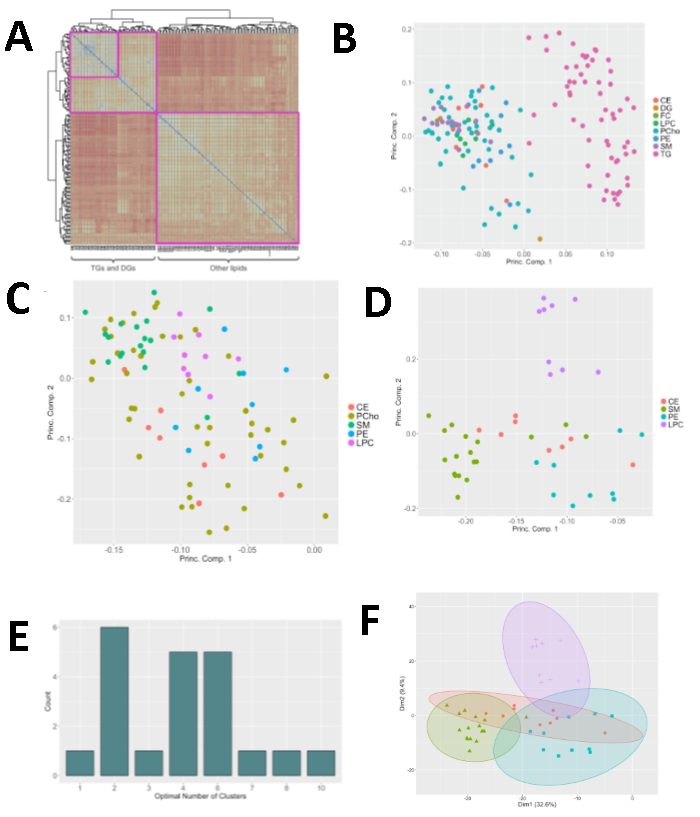
**

**Supplementary Figure 1: Clustering tendencies for the different lipid sub-classes. A.** Hierarchical clustering of all lipid species detected in positive ion mode. Visual assessment of clustering tendency indicates a bifurcation for the Fenland dataset. The triacylglycerol subgroup appears to contain further substructure. **B.** Principal components analysis (PCA) of the lipid species detected in positive ion mode. Key: CE cholesterol ester, SM sphingomyelin, PE phosphatidylethanolamine, LPC lysophosphatidylcholine, PC phosphatidylcholine, FC free cholesterol, TG triacylglycerol, DG diacylglycerol. **C.** As **B** but excluding triacylglycerols and diacylglyecrols. **D**. As **C** but excluding phosphatidylcholines. **E.** Indices for estimating the optimal number of clusters for CEs, LPCs, Pes and SMs do not give a clear value for the Fenland Dataset in self organising maps. **F.** K-means clustering (k=4) of CEs, LPCs, Pes, and SMs confirms clustering according to lipid class (Fenland dataset). Colours indicate cluster membership.

**Supplementary Table 1:** Hierarchical, k-means and SOM clustering of cholesterol esters (CEs), lysophosphatidylcholines (LPCs), phosphatidylethanolamines (PEs) and sphingomyelins (SMs) produce varies results in the Fenland lipidomic dataset.


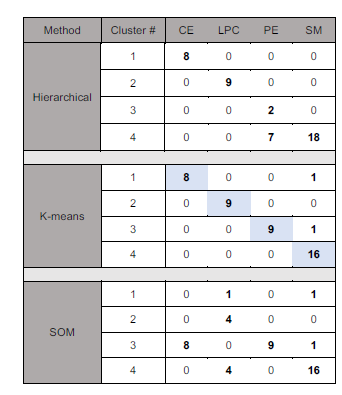


*
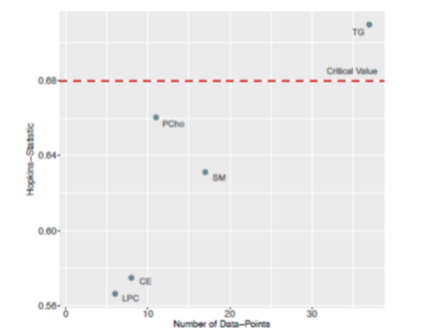
*

**Supplementary Figure 2:** Hopkin’s statistics of individual lipid classes confirm that only triacylglycerols have significant sub-structure in the NSHD cohort. Key: LPC lysophosphatidylcholines, CE cholesterol esters, PCho phosphatidylcholines, SM sphingomyelins, TG triacylglycerols.

*Limiting clustering of TAGs to common species further highlights the similarities between the Fenland and NSHD cohorts*

As in general more TAG species were found in samples from the NSHD cohort, the clustering used in **figure 3** was re-performed on the 25 common TAG species detected in both the Fenland and NSHD cohorts. This produced similar clusterings between the two cohorts (**Supplementary Figure 3a & 3b**) with cluster 3 sharing 5 TAGs out of 6 detected in both the Fenland and NSHD cohorts (**Supplementary Figure 3c**).


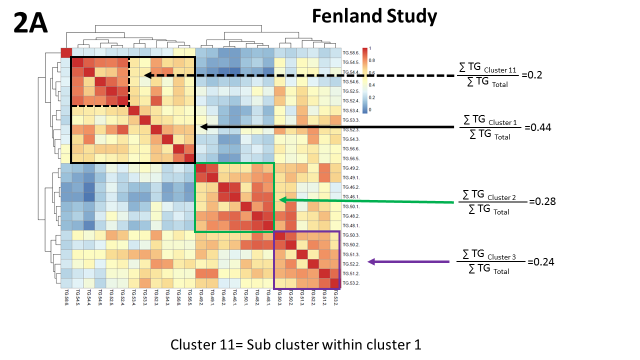


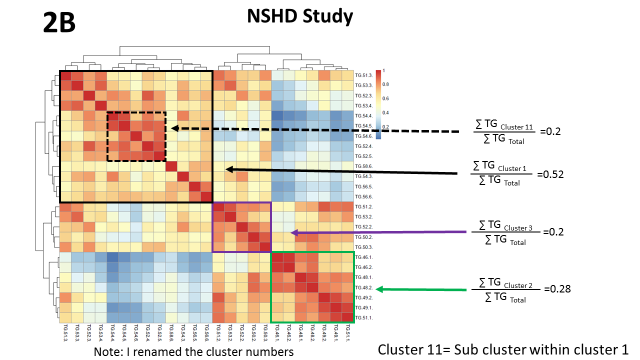


*
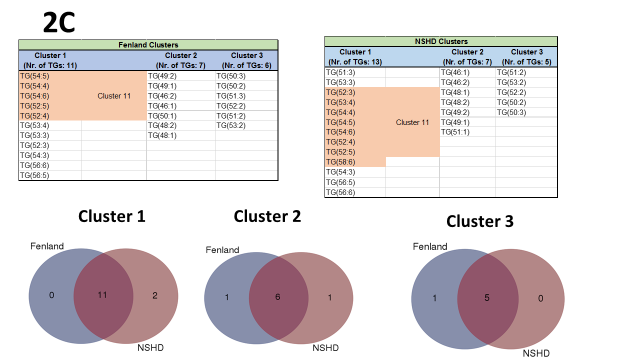
*

**Supplementary Figure 3: A:** Heat map of the Bayesian Hierarchical Cluster Analysis (B-HCA) for the Fenland cohort using only triacylglycerols (TAGs) detected in both cohort studies. **B.** Heat map opf the Bayesian Hierarchical Cluster Analysis (B-HCA) for the NSHD cohort using only triacylglycerols detected in both cohort studies. C: Tables of TAGs detected and Venn Diagram for the overlap between TAGs found in difference clusters across the two cohort studies.

*Predicting hepatic steatosis in a sub-cohort of Fenland*

To further explore the predictive capability of the data, an OPLS-DA model was built using spectra from individuals where only a FLI was calculated and no ultrasound measurement was present (number of samples = 566; R^2^(X)=63%, R^2^(Y)=52% , Q^2^=46%; ANOVA-CV (p<10^-6^); **Supplementary Figure 4a**-**4b**). These predictions were represented as a ROC with an AUC of 0.93 (84.8% correct prediction of class status) **(Supplementary Figure 4c**). This model was then used to confirm the status of fatty liver in the group where both ultrasound and fatty liver index were available, using the ultrasound measurements for the classification of disease presence. This produced a ROC curve with an AUC of 0.87 for the 855 samples used in the test set (equivalent to 78.5% correct prediction) (**Supplementary Figure 4d**). Predictions were more accurate for fatty liver disease, and misclassifications were more often at the border in terms of FLI between classifications.

*
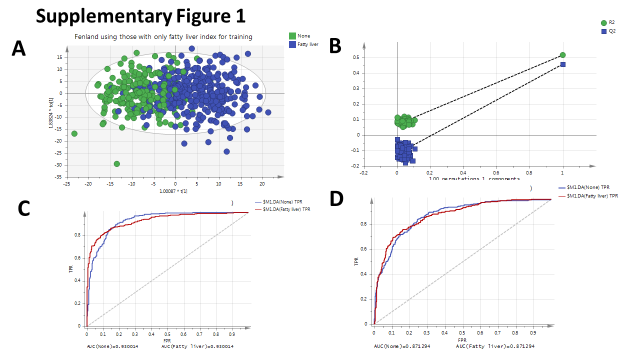
*

*
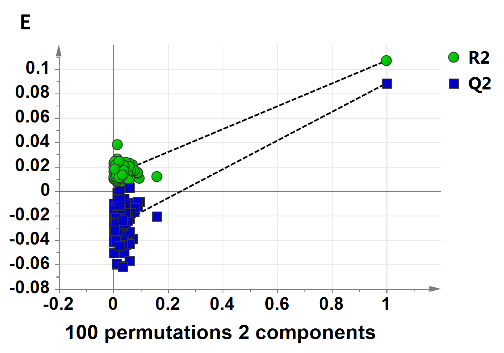
*

**Supplementary Figure 4: A:** OPLS-DA model built using samples from individuals where only the Fatty Liver Index (FLI) was present to predict hepatic steatosis (train set). A robust model was built that readily discriminated those with (blue) and without (green) hepatic steatosis. **B.** Permutation test analysis to assess the robustness of the model. **C.** ROC curves for prediction of the samples used to build the model. **D.** ROC curves for the prediction of those with hepatic steatosis as confirmed by ultrasound (test set). **E.** Permutation test analysis to assess the robustness of the model examining changes in TAG profile according to hepatic steatosis status as determined by ultrasound.

*LCMS demonstrates a shift in the TAG profile toward shorter chain more saturated FA containing TGs paralleling an increase in DNL in wildtype mice*

Intact hepatic TGs were analysed using LCMS to assess for any effect of diet or genotype on the TG profile, and whether these changes correlated with the incorporation of *de novo* synthesised fatty acids into TAGs and the rate of DNL. Wild type mice were also compared between HFD and RCD using PLS-DA (R^2^ = 0.969, Q^2^ = 0.815), which passed cross-validation by random permutations and CV-ANOVA (p = 0.0104). The wild type mice did not demonstrate a clear dichotomy in TGs between longer chain more unsaturated TAGs and shorter chain more saturated TGs between the two diets (**Supplementary Figure 5**).


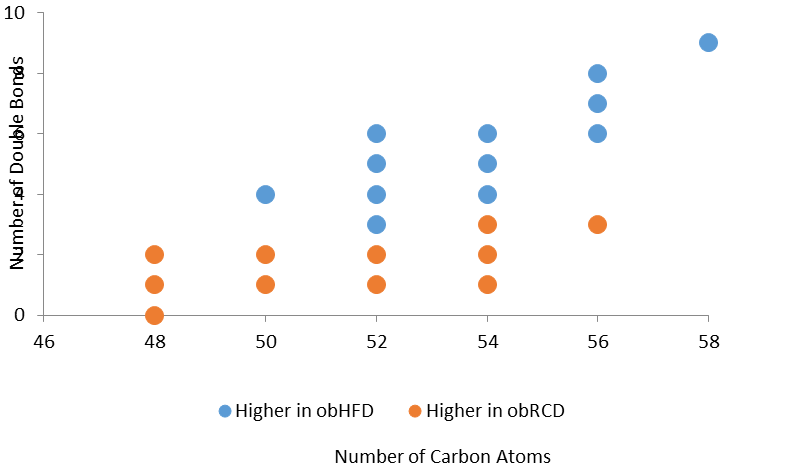

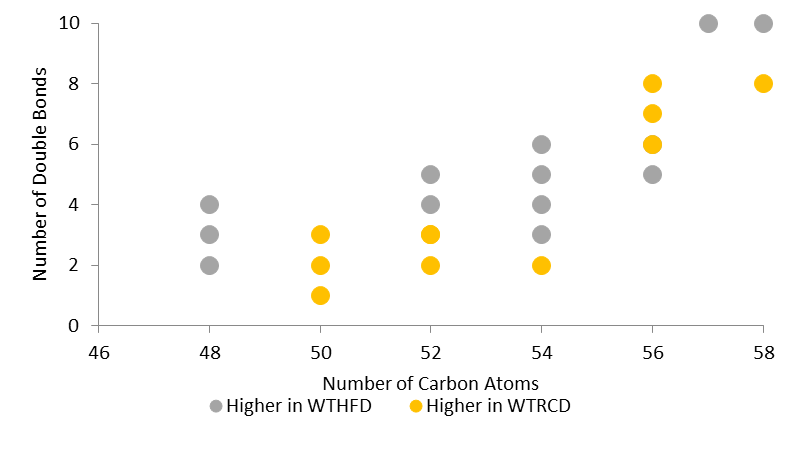


***Supplementary Figure 5: TGs plotted according to carbon number and double bond number comparing between high fat diet in ob/ob mice* [A] *and in wild type mice* [B]*.*** *The hepatic TAG profile in HFD ob/ob mice (obHFD) were compared to RCD fed ob/ob mice (obRCD) using PLS-DA, with a VIP > 1 being taken as significant. This resulted in increased TGs containing shorter chain more saturated FAs in obRCD compared to obHFD.* **[B]** *The wild type mice fed RCD (WTRCD) did not have a dichotomous difference between longer chain more unsaturated TAGs and shorter more saturated TGs when compared to wild type mice fed HFD (WTHFD) using PLS-DA.*

*
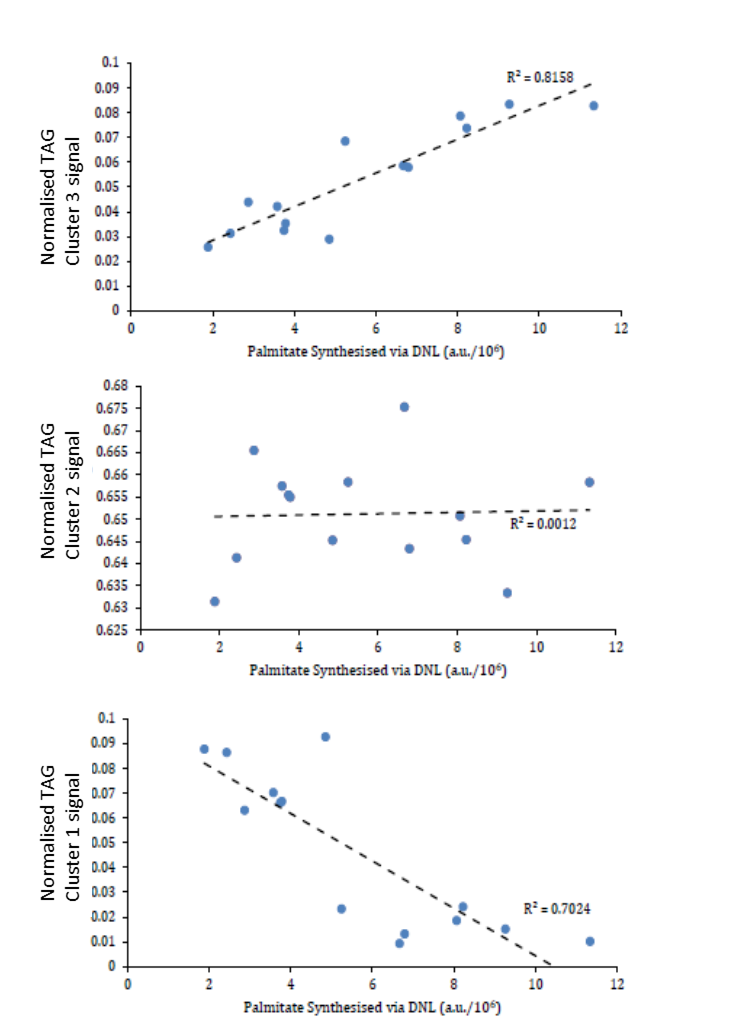
*

**Supplementary Figure 6:** Simple linear modelling of putative TAG clusters against de novo palmitate synthesised in mice. **[A]** TAG Fenland cluster 3 shows significant positive correlation with the hepatic palmitate synthesised via DNL (R^2^ = 0.81581, p < 1*10^-5^) in ob/ob mice on a chow diet. **[B]** TAG Fenland cluster 2 summated signal shows no correlation with hepatic palmitate synthesised via DNL (R^2^ = 0.00123, p = 0.9054). **[C]** TAG Fenland cluster 1 shows a significant negative correlation with quantity of palmitate synthesised via DNL (R^2^ = 0.70236, p < 5*10^-4^).

*RT-PCR indicates that the dietary intervention induced changes in the expression of the lipogenic gene network in mice on a chow diet compared with those on a high fat diet*

Acaca encodes acetyl CoA carboxylase (ACC) 1, which decreased in expression on a high fat diet compared with a normal chow diet in both *ob/ob* and wildtype mice (**Supplementary Figure 6***;* p < 0.001, n= 7 for ob/ob comparison); it is also increased in the *ob/ob* mice fed regular chow diet compared to the wild type mice (**Supplementary Figure 6***;* p < 0.001, n= 6). Additionally, acacb, which encodes a different isoform of the same enzyme, ACC2, is also significantly more expressed in *ob/ob* mice on regular chow compared to those on the high fat diet (p < 0.0001, n = 7), and ACACB was expressed more in these *ob/ob* mice on regular chow than the wild type mice fed the same diet (p < 0.0001, n = 6). Furthermore, Fasn, the gene for fatty acid synthase, is significantly dwonregulated in *ob/ob* mice on the high fat diet compared to those on the regular chow diet (p < 0.01, n = 7). Fasn is also upregulated in *ob/ob* mice compared to wild type mice both fed the regular chow diet (p < 0.0001, n = 6) or when

***

***

****

****

*

****

****

***Supplementary Figure 7: Expression of Acaca, Elovl6 and Scd1, three principle enzymes within the lipogenic network.*** *Acaca is crucial to the initiation of lipogenesis and is significantly increased in obob mice fed a regular chow diet (obRCD) compared to those fed a high fat diet (obHFD), as well as wild type mice also fed RCD (WTRCD). Elovl6, a principal elongase, is also significantly increased in obRCD compared to obHFD and WTRCD. Wild type mice fed HFD (WTHFD), also have a significantly lower Elovl6 expression than obHFD. obRCD expressed Scd1, a crucial fatty acid desaturase, significantly more than obHFD or WTRCD. All data is represented as mean ± SEM analysed by two-way ANOVA and Tukey’s multiple comparison test.*

**** p < 0.0001

*** p < 0.001

** p < 0.01

* p < 0.05

both genotypes were fed high fat diet (p < 0.001, n = 6). The elongase, Elovl6, and the desaturase, Scd1, were significantly decreased in *ob/ob* mice fed the high fat diet than *ob/ob* mice fed chow diet (**Supplementary Figure 7***;* p < 0.0001, n = 7). Both Elovl6 and Scd1 were significantly increased in *ob/ob* mice compared to wild type mice when they consumed the regular chow diet (*Fig 3.4*; p < 0.0001, n = 6). Elovl6 is significantly increased in *ob/ob* mice fed the high fat diet compared to wild type mice fed the same diet (**Supplementary Figure 7**; p < 0.05, n = 6). There was, however, no significant difference in either Srebf1 or Mlxipl, the genes for SREBP1c and ChREBP respectively, between the mice when classified either by genotype or diet.


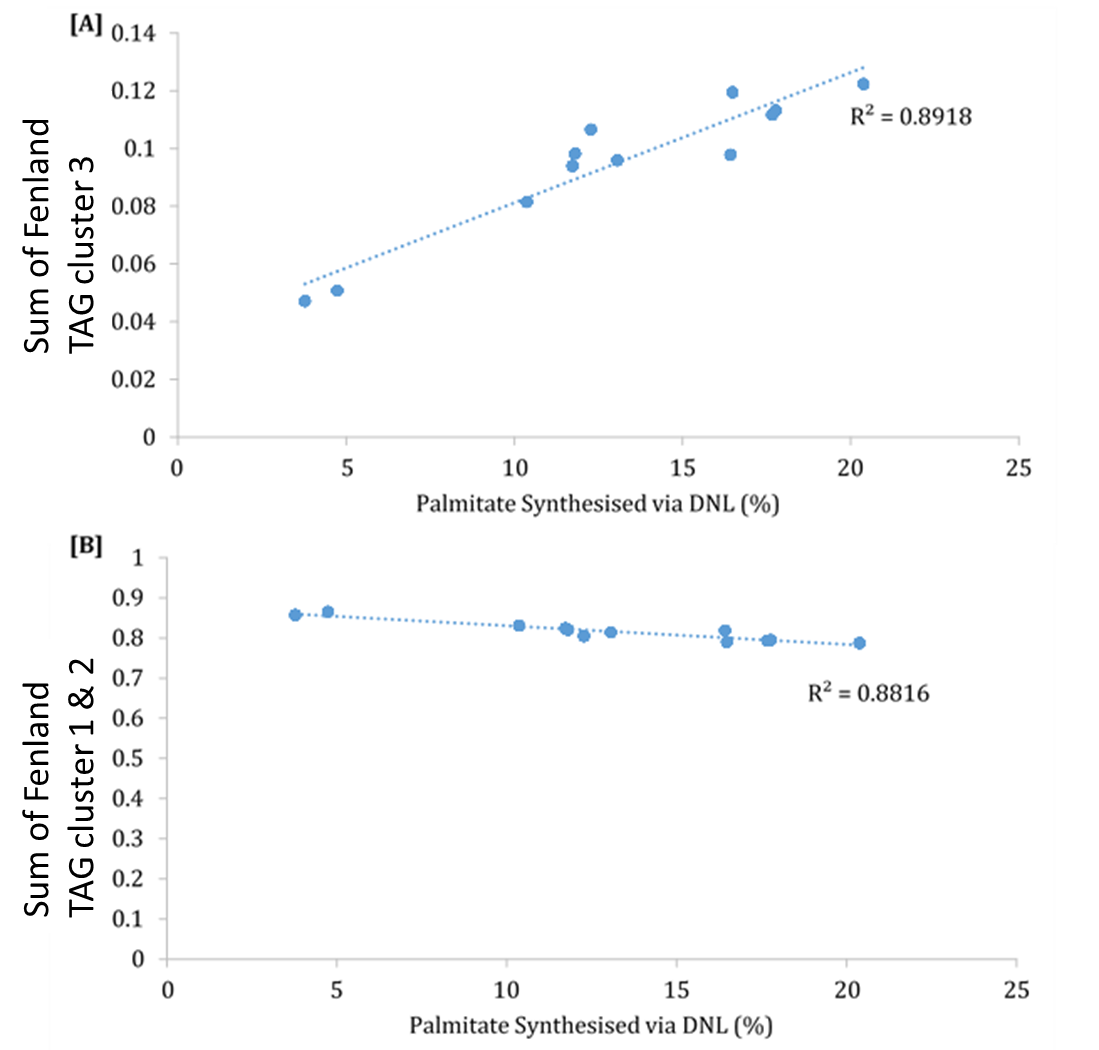


**Supplementary Figure 8: Simple linear modelling of Fenland TAG cluster 3 [A] and TAG Fenland cluster 1 and 2 [B] against the quantity of palmitate synthesised via DNL calculated, using deuterium incorporation, during a day of high carbohydrate feeding.** [A] A panel of TGs with FA moieties with a low carbon and double bond number, TAG Fenland cluster 1, demonstrates a significant positive correlation with the quantity of palmitate synthesised via DNL when measured using the standard protocol of incorporation of deuterium. [B] Fenland TAG clusters 1 and 2, which comprises TGs containing longer chain more unsaturated TGs, demonstrates a significant negative correlation with DNL measured by deuterium incorporation into palmitate.

*The association of other risk factors with the TAG cluster containing saturated FAs (Fenland)*

To investigate further the TAGs associated with DNL, we performed a combination of linear regression and logistic regression of these factors with a range of other explanatory variables measured in the Fenland study (**Supplementary Figure 9**).


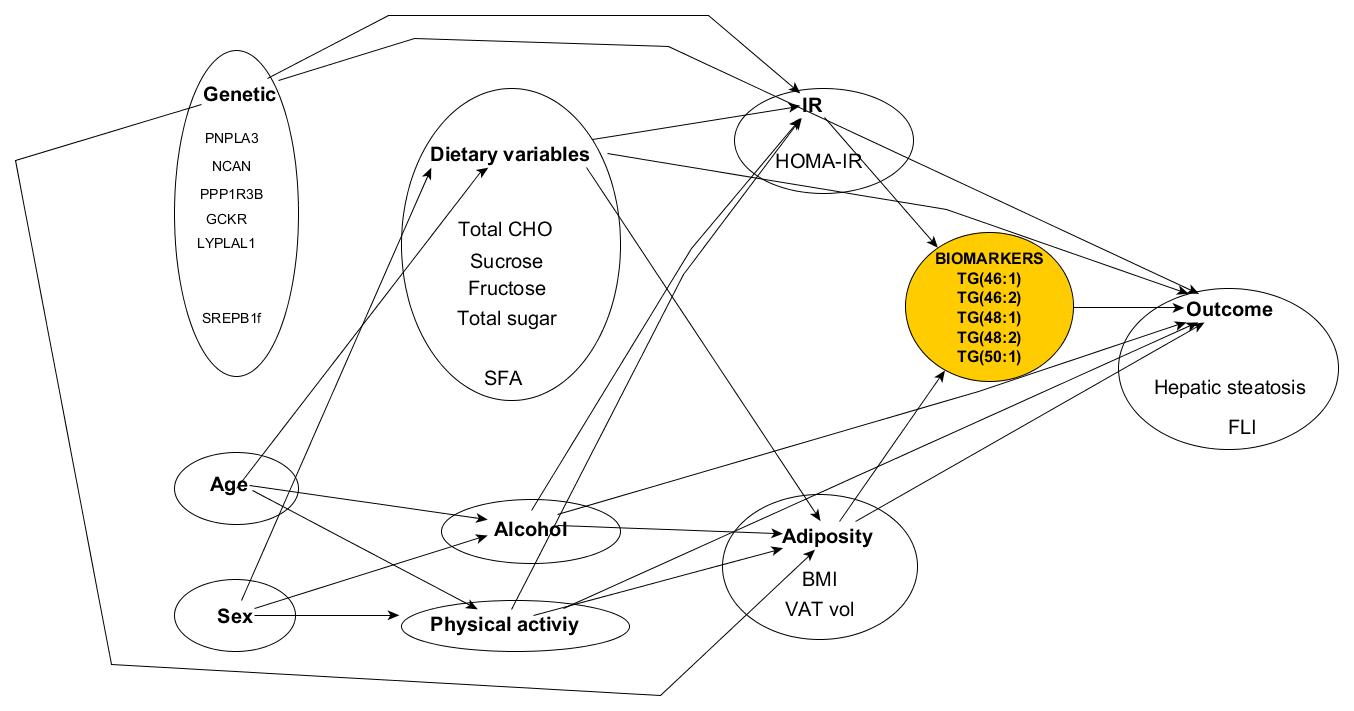


**Supplementary Figure 9:** In order to explore if these TG markers might be markers of hepatic steatosis and *de novo* lipogenesis we explored the association between these markers and liver fat as assessed by ultrasound. The liver score is assigned according to indications of normal (≤4) or mild (5-7), moderate (8-10) or severe liver steatosis (≥11) according to increased echo reflectivity of the liver parenchyma (bright liver in comparison with the kidney); decreased visualisation of the intra-hepatic vasculature; and attenuation of the ultrasound beam. The covariates and confounding factors were built up according to our hypothetical model of factors impacting on hepatic steatosis. We built up the models from the left: intrinsic characteristics, followed by lifestyle characteristics, followed by metabolic perturbations. In order to explore which variables impacted on the relationship between the markers and steatosis we retained all the variables while building up the models regardless of whether they were significant.

**Supplementary Table 1: Logistic regression of key triglycerides with fatty liver disease as measured by fatty liver index (FLI).** Each model is performed by linear regression with the outcome FLI and the triglyceride markers as predictors. The triglyceride markers were all log(n) transformed except 46:2 which was log(10) transformed such that the residuals approximated to a normal distribution. The simple model is adjusted for age, sex, total triglyceride and analysis batch. Model 2 is the simple model with adjustments for PAEE, diet (proportion SFA, proportion of fructose, and proportion of free sugar), smoking and genetic factors (SNPs: rs780094, rs4240624, rs11868035, rs2228603, rs738409). Model 3 is model 2 additionally adjusted for log(n) alcohol intake (g/d). Model 4 is model 3 additionally adjusted for log(n) HOMA-IR. Model 5 is model 4 additionally adjusted for log(n) BMI. Model 6 is model 4 additionally adjusted for the √VAT volume. Model 7 is model 4 additionally adjusted for waist circumference. Participants who self-reported to be taking lipid medication were excluded (n=20), participants who were designated not plausible reporters for dietary data were also excluded (n=79).

| **TAG species** | **46:1** | **46:2** | **48:1** | **48:2** | **50:1** |
| --- | --- | --- | --- | --- | --- |
| **Simple model** | 6.30 ± 0.94 (0.000) | 5.55 ± 1.55 (0.000) | 11.2 ± 1.02 (0.000) | 10.2 ± 1.13 (0.000) | 17.8 ± 1.32 (0.000) |
| **Model 2** | 5.66 ± 1.04 (0.000) | 4.58 ± 1.73 (0.008) | 10.5 ± 1.12 (0.000) | 10.1 ± 1.23 (0.000) | 16.3 ± 1.46 (0.000) |
| **Model 3** | 6.02 ± 1.14 (0.000) | 5.20 ± 1.90 (0.006) | 10.3 ± 1.22 (0.000) | 9.61 ± 1.33 (0.000) | 17.2 ± 1.62 (0.000) |
| **Model 4** | 2.83 ± 1.00 (0.005) | 1.45 ± 1.64 (0.379) | 5.66 ± 1.10 (0.000) | 5.32 ± 1.19 (0.000) | 11.0 ± 1.48 (0.000) |
| **Model 5** | 2.78 ± 0.62 (0.000) | 2.26 ± 1.02 (0.027) | 4.77 ± 0.68 (0.000) | 4.39 ± 0.74 (0.000) | 8.24 ± 0.92 (0.000) |
| **Model 6** | 1.57 ± 0.77 (0.043) | 1.54 ± 1.24 (0.212) | 3.47 ± 0.86 (0.000) | 3.64 ± 0.92 (0.000) | 5.72 ± 1.18 (0.000) |
| **Model 7** | 2.09 ± 0.61 (0.001) | 1.18 ± 1.00 (0.238) | 3.81 ± 0.68 (0.000) | 3.69 ± 0.73 (0.000) | 7.03 ± 0.91 (0.000) |

Observations:

- 48 and 50 length TGs are associated with FLI after adjustment for all covariates.
- 46 length TGs are associated with FLI adjusted for BMI but not after adjustment for waist or VAT vol.
- There was a consistent pattern of the effect of covariates alcohol intake and HOMA-IR on the coefficients between analysis of FLI and liver score.
- Using waist or VAT volume makes little difference.

***Supplementary Table 2: Association between*** rs4240624 (PPP1R3B) and specific TAG species.

|  | **TAG46:1** | **TAG46:2** | **TAG48:1** | **TAG48:2** | **TAG50:1** |
| --- | --- | --- | --- | --- | --- |
| Model 1 | -0.15 ± 0.05  (0.001) | -0.07 ± 0.03  (0.01) | -0.13 ± 0.04  (0.001) | -0.11 ± 0.04  (0.002) | -0.08 ± 0.03  (0.01) |
| Model 2 | -0.14 ± 0.05  (0.002) | -0.07 ± 0.03  (0.01) | -0.13 ± 0.04  (0.002) | -0.11 ± 0.04  (0.003) | -0.07 ± 0.03  (0.02) |
| Model 3 | -0.14 ± 0.05  (0.002) | -0.07 ± 0.03  (0.01) | -0.12 ± 0.04  (0.002) | -0.11 ± 0.04  (0.003) | -0.07 ± 0.03  (0.02) |

Model 1 is the association between SNP and each of the TG markers assessed by linear regression analysis. The analysis was adjusted for total triglycerides and batch number. Model 2 is model 1 but with additional adjustment for age and sex. Model 3 is model 2 additionally adjusted for log (n) BMI.

All TG markers were log (n) transformed for analysis and the untransformed results are presented.

SNP rs4240624 (PPP1R3B) was strongly associated with all the TG markers and was unaffected by adjustment for age, sex or BMI. No other hepatic steatosis-related SNP tested was associated with the TG markers (when adjusted for total triglycerides).

***Supplementary Table 3: Proportion of total carbohydrate in the diet***

|  | 46:1 | 46:2 | 48:1 | 48:2 | 50:1 |
| --- | --- | --- | --- | --- | --- |
| Model 1 | 0.000 ± 0.003  (0.795) | 0.000 ± 0.002  (0.892) | -0.003 ± 0.003  (0.229) | 0.003 ± 0.002  (0.257) | -0.005 ± 0.002  (0.027) |
| Model 2 | 0.000 ± 0.003  (0.898) | 0.000 ± 0.002  (0.894) | -0.003 ± 0.003  (0.347) | 0.003 ± 0.002  (0.220) | -0.004 ± 0.002  (0.073) |
| Model 3 | 0.000 ± 0.003  (0.972) | 0.000 ± 0.002  (0.825) | -0.002 ± 0.003  (0.419) | 0.003 ± 0.002  (0.173) | -0.003 ± 0.002  (0.097) |
| Model 4 | 0.001 ± 0.003  (0.668) | 0.001 ± 0.002  (0.675) | -0.000 ± 0.003  (0.875) | 0.005 ± 0.002  (0.062) | -0.002 ± 0.002  (0.409) |
| Model 5 | 0.000 ± 0.003  (0.962) | 0.000 ± 0.002  (0.921) | -0.002 ± 0.003  (0.508) | 0.003 ± 0.002  (0.152) | -0.002 ± 0.002  (0.212) |

Model 1 is unadjusted apart from analysis batch; Model 2 is model 1 adjusted for age and sex; Model 3 is model 2 additionally adjusted for log (n) BMI; Model 4 is model 2 additionally adjusted for √visceral adipose volume; Model 5 is model 4 additionally adjusted for log (n) HOMA-IR. All TG markers were log (n) transformed for analysis apart from TG46:2 which was log (10) transformed. The transformed results are presented.

Analysis repeated using absolute carbohydrate load (not as a proportion of total energy). There was no association apart from TG50:1 fully adjusted model -0.094 ± 0.039 (P=0.016).

So none of the TG markers are modulated by the total habitual carbohydrate load or proportion of carbohydrate in the diet.

**Supplementary Table 4: Proportion of total fat in the diet**

|  | 46:1 | 46:2 | 48:1 | 48:2 | 50:1 |
| --- | --- | --- | --- | --- | --- |
| Model 1 | 0.004 ± 0.003  (0.273) | 0.002 ± 0.002  (0.336) | 0.000 ± 0.003  (0.897) | 0.000 ± 0.003  (0.896) | 0.002 ± 0.002  (0.375) |
| Model 2 | 0.003 ± 0.003  (0.334) | 0.002 ± 0.002  (0.323) | 0.000 ± 0.003  (0.914) | 0.000 ± 0.003  (0.922) | 0.000 ± 0.002  (0.828) |
| Model 3 | 0.002 ± 0.003  (0.546) | 0.002 ± 0.002  (0.346) | 0.000 ± 0.003  (0.903) | 0.000 ± 0.003  (0.914) | 0.000 ± 0.002  (0.83) |
| Model 4 | 0.001 ± 0.003  (0.743) | 0.002 ± 0.002  (0.437) | -0.002 ± 0.003  (0.493) | -0.001 ± 0.003  (0.594) | -0.001 ± 0.002  (0.604) |
| Model 5 | 0.002 ± 0.002  (0.396) | 0.001 ± 0.002  (0.631) | -0.003 ± 0.003  (0.321) | -0.002 ± 0.003  (0.720) | -0.002 ± 0.002  (0.418) |

Model 1 is unadjusted apart from analysis batch; Model 2 is model 1 adjusted for age and sex; Model 3 is model 2 additionally adjusted for log (n) BMI; Model 4 is model 2 additionally adjusted for √visceral adipose volume; Model 5 is model 4 additionally adjusted for log (n) HOMA-IR. All TG markers were log (n) transformed for analysis apart from TG46:2 which was log (10) transformed. The transformed results are presented.

Repeated analysis for absolute dietary fat load. There was no association apart from TG50:1 in the fully adjusted model -0.067 ± 0.033 (P=0.042)

TG markers are not modulated by habitual fat load or the proportion of total fat in the diet.

**Supplementary Table 5: Proportion of saturated fat in the diet**

|  | 46:1 | 46:2 | 48:1 | 48:2 | 50:1 |
| --- | --- | --- | --- | --- | --- |
| Model 1 | 0.022 ± 0.007  (0.001) | 0.013 ± 0.004  (0.002) | 0.023 ± 0.006  (0.000) | 0.018 ± 0.005  (0.001) | 0.017 ± 0.004  (0.000) |
| Model 2 | 0.022 ± 0.007  (0.001) | 0.013 ± 0.004  (0.002) | 0.022 ± 0.006  (0.000) | 0.019 ± 0.005  (0.000) | 0.014 ± 0.004  (0.001) |
| Model 3 | 0.021 ± 0.007  (0.001) | 0.013 ± 0.004  (0.002) | 0.021 ± 0.006  (0.000) | 0.018 ± 0.005  (0.001) | 0.013 ± 0.004  (0.002) |
| Model 4 | 0.019 ± 0.007  (0.003) | 0.012 ± 0.004  (0.003) | 0.018 ± 0.006  (0.001) | 0.016 ± 0.005  (0.001) | 0.011 ± 0.004  (0.009) |
| Model 5 | 0.017 ± 0.006  (0.007) | 0.011 ± 0.004  (0.008) | 0.016 ± 0.006  (0.004) | 0.015 ± 0.005  (0.004) | 0.009 ± 0.004  (0.024) |

Model 1 is unadjusted apart from analysis batch; Model 2 is model 1 adjusted for age and sex; Model 3 is model 2 additionally adjusted for log (n) BMI; Model 4 is model 2 additionally adjusted for √visceral adipose volume; Model 5 is model 4 additionally adjusted for log (n) HOMA-IR. All TG markers were log (n) transformed for analysis apart from TG46:2 which was log (10) transformed. The transformed results are presented.

Repeated analysis for absolute SFA load. There were no associations apart from TG48:1 [0.082 ± 0.040 (P=0.039)] and TG50:1 [0.081 ± 0.03 (0.008)] in the unadjusted models. There was no association with the fully adjusted model.

The proportion of saturated fat in the habitual diet is strongly positively associated with all the TG markers. This association is mostly robust for adjustment for HOMA-IR, BMI and visceral adipose volume, indicating the association is via a mechanism not governed by adiposity or insulin sensitivity. The habitual saturated fat load also has an impact on some but not all of the markers.

**Supplementary Table 6: Proportion of free sugar in the diet**

|  | 46:1 | 46:2 | 48:1 | 48:2 | 50:1 |
| --- | --- | --- | --- | --- | --- |
| Model 1 | 0.008 ± 0.004  (0.04) | 0.005 ± 0.003  (0.071) | 0.007 ± 0.003  (0.040) | 0.007 ± 0.003  (0.018) | 0.008 ± 0.003  (0.002) |
| Model 2 | 0.008 ± 0.004  (0.040) | 0.005 ± 0.003  (0.065) | 0.007 ± 0.003  (0.049) | 0.008 ± 0.003  (0.011) | 0.007 ± 0.003  (0.011) |
| Model 3 | 0.008 ± 0.004  (0.053) | 0.005 ± 0.003  (0.074) | 0.006 ± 0.003  (0.086) | 0.007 ± 0.003  (0.020) | 0.006 ± 0.002  (0.024) |
| Model 4 | 0.007 ± 0.004  (0.079) | 0.004 ± 0.003  (0.091) | 0.005 ± 0.003  (0.140) | 0.007 ± 0.003  (0.031) | 0.005 ± 0.003  (0.052) |
| Model 5 | 0.005 ± 0.004  (0.171) | 0.003 ± 0.003  (0.187) | 0.003 ± 0.003  (0.35) | 0.005 ± 0.003  (0.089) | 0.004 ± 0.002  (0.131) |

Model 1 is unadjusted apart from analysis batch; Model 2 is model 1 adjusted for age and sex; Model 3 is model 2 additionally adjusted for log (n) BMI; Model 4 is model 2 additionally adjusted for √visceral adipose volume; Model 5 is model 4 additionally adjusted for log (n) HOMA-IR. All TG markers were log (n) transformed for analysis apart from TG46:2 which was log (10) transformed. The transformed results are presented.

Repeated analysis for absolute free sugar load. There were no associations apart from TG50:1 [0.055 ± 0.023 (0.018)] in unadjusted model.

There was an association between proportion of free sugar in the habitual diet and most of the TG markers. This association was partly mediated by adiposity and insulin sensitivity. This association was not seen for absolute free sugar load in the habitual diet, only as a proportion of total energy.

**Supplementary References:**

1. Burgoine T, Forouhi, N.G., Griffin, S.J., Wareham, N.J., Monsivais, P. (2014) Associations between exposure to takeaway food outlets, takeaway food consumption, and body weight in Cambridgeshire, UK: population based, cross sectional study. BMJ 348: g1464.

2. Mulligan AA, Luben, R.N., Bhaniani, A., Parry-Smith, D.J., O'Connor, L., Khawaja, A.P., Forouhi, N.G., Khaw, K.T.; EPIC-Norfolk FFQ Study. (2014) A new tool for converting food frequency questionnaire data into nutrient and food group values: FETA research methods and availability. BMJ Open 4: e004503.

3. Bedogni G, Bellentani, S., Miglioli, L., Masutti, F., Passalacqua, M., Castiglione, A., Tiribelli, C. (2006) The Fatty Liver Index: a simple and accurate predictor of hepatic steatosis in the general population. BMC Gastroenterol 6: 33.

4. Speliotes EK, Yerges-Armstrong, L.M., Wu, J., Hernaez, R., Kim, L.J., Palmer, C.D., Gudnason, V., Eiriksdottir, G., Garcia, M.E., Launer, L.J., Nalls, M.A., Clark, J.M., Mitchell, B.D., Shuldiner, A.R., Butler, J.L., Tomas, M., Hoffmann, U., Hwang, S.J., Massaro, J.M., O'Donnell, C.J., Sahani, D.V., Salomaa, V., Schadt, E.E., Schwartz, S.M., Siscovick, D.S.; NASH CRN; GIANT Consortium; MAGIC Investigators, Voight, B.F., Carr, J.J., Feitosa, M.F., Harris, T.B., Fox, C.S., Smith, A.V., Kao, W.H., Hirschhorn, J.N., Borecki, I.B.; GOLD Consortium. (2011) Genome-wide association analysis identifies variants associated with nonalcoholic fatty liver disease that have distinct effects on metabolic traits. PLoS Genet 7: e1001324.

5. Eiden M, Koulman, A., Hatunic, M., West, J.A., Murfitt, S., Osei, M., Adams, C., Wang, X., Chu, Y., Marney, L., Roberts, L.D., O'Rahilly, S., Semple, R.K., Savage, D.B., Griffin, J.L. (2015) Mechanistic insights revealed by lipid profiling in monogenic insulin resistance syndromes. Genome Med 7: 63.

6. Lawson RG, Jurs, P.C. (1990) New index or clustering tendency and its application to chemical problems. J Chem Inf Model 30: 36-41.
